# An algorithm to predict the connectome of neural microcircuits

**DOI:** 10.3389/fncom.2015.00120

**Published:** 2015-10-08

**Authors:** Michael W. Reimann, James G. King, Eilif B. Muller, Srikanth Ramaswamy, Henry Markram

**Affiliations:** Blue Brain Project, École Polytechnique Fédérale de Lausanne (EPFL) Biotech CampusGeneva, Switzerland

**Keywords:** neocortex, algorithm development, connectome mapping, synaptic transmission, *in silico*, somatosensory cortex, cortical circuits

## Abstract

Experimentally mapping synaptic connections, in terms of the numbers and locations of their synapses and estimating connection probabilities, is still not a tractable task, even for small volumes of tissue. In fact, the six layers of the neocortex contain thousands of unique types of synaptic connections between the many different types of neurons, of which only a handful have been characterized experimentally. Here we present a theoretical framework and a data-driven algorithmic strategy to digitally reconstruct the complete synaptic connectivity between the different types of neurons in a small well-defined volume of tissue—the micro-scale connectome of a neural microcircuit. By enforcing a set of established principles of synaptic connectivity, and leveraging interdependencies between fundamental properties of neural microcircuits to constrain the reconstructed connectivity, the algorithm yields three parameters per connection type that predict the anatomy of all types of biologically viable synaptic connections. The predictions reproduce a spectrum of experimental data on synaptic connectivity not used by the algorithm. We conclude that an algorithmic approach to the connectome can serve as a tool to accelerate experimental mapping, indicating the minimal dataset required to make useful predictions, identifying the datasets required to improve their accuracy, testing the feasibility of experimental measurements, and making it possible to test hypotheses of synaptic connectivity.

## Introduction

The connectome is a key determinant of the computational capability and capacity of the brain (Chklovskii et al., 2004; Hofer et al., 2009; Seung, 2012). In a spatial region where all local neurons can potentially interact monosynaptically, the activity of each individual neuron is shaped by the spatio-temporal pattern of activation of its input synapses, and its impact on other neurons is determined by the synapses it forms on its many targets. Mapping neurons' input and output synapses is therefore fundamental to understanding their function in neural microcircuitry, and ultimately, the functional role of each type of neuron in the brain. However, mapping all the synapses formed between all the neurons in the brain is still a technically insurmountable challenge, which becomes even more extreme if one considers the importance of variations between individuals, species, and genders, and the changes associated with different stages of development. Furthermore, while electron microscopy (EM) combined with automated or semi-automated reconstruction techniques (Denk and Horstmann, 2004; Chklovskii et al., 2010; Kleinfeld et al., 2011) makes it possible to characterize the anatomy of synaptic connections in increasingly large volumes of neural tissue, no currently available technique can characterize the synapses formed by neurons belonging to different electrophysiological types.

There is a long tradition of studies exploring the statistics of connectivity in an attempt to identify general organizing rules that predict connectivity. Early findings that thalamus innervates layer 4 of the cortex without targeting specific neuron types (Peters and Feldman, 1976; Peters et al., 1979) have been generalized in rules stating that connections are untargeted, that the fraction of axo-dendritic appositions forming synapses is constant, and that most connections are formed of only one synapse (Braitenberg and Schüz, 1998; Braitenberg, 2001). Early attempts to predict intracortical connectivity, using these rules, did not take account of the specific morphology of axonal and dendritic arborizations and, while later approaches improved on these attempts by using reconstructed arbors, they still concluded that most connections consist of a single synapse (Hellwig, 2000). However, all studies of synaptically coupled pairs of neurons in the neocortex report multiple synapses (Deuchars et al., 1994; Markam et al., 1997; Markram et al., 2004; Feldmeyer et al., 1999, 2006; Reyes and Sakmann, 1999; Wang et al., 2002; Silver et al., 2003; Silberberg and Markram, 2007; Frick et al., 2008), and (Shepherd et al., 2005) found that the mean number of synapses between cell pairs is proportional to the axo-dendritic overlap. Fares and Stepanyants (2009) have therefore proposed an algorithm that includes a step to explicitly remove structurally weak connections (i.e., connections with too few synapses).

These studies were handicapped by the lack of data on the cellular composition of neural tissue (i.e., neuron densities and the proportions of neurons belonging to different morphological types or *m-types*). However, a recent draft digital reconstruction of a neural microcircuit (height: 2082 μm; diameter: 460 μm), in the somatosensory cortex of a P14 Wistar rat, identifies 55 layer-specific and morphologically distinct types of neurons, as well as 207 morpho-electrical types (Markram et al., 2015). This implies that there could be as many as 3025 (55^2^) unique types of connections between neurons belonging to different m-types and 42,849 (207^2^) between morpho-electrical types, of which only a negligible number have been experimentally characterized. The reconstruction also provides an estimate of the total number of neurons (~32,000), and the layer-wise densities and numbers for each type of neuron—its neuronal composition.

On the assumption that the neuronal morphologies and neuronal composition are complete, we developed a theoretical framework and a data-driven algorithm to predictively reconstruct the micro-scale connectome. The algorithm implements established principles of connectivity (e.g., all connections in neocortex involve multiple synapses, synapse locations are largely determined by the incidental apposition between neuronal arbors) and leverages interdependencies between fundamental microcircuit properties (e.g., numbers of synapses/connection, bouton densities) to constrain its predictions. The algorithm is also based on a set of logical arguments that can be invoked when the neuronal composition is provided: (1) Since all possible postsynaptic targets are present, all synapses formed between the neurons of the microcircuit (intrinsic synapses) are also necessarily present; (2) since the total number of synapses can be estimated from the number of boutons, the bouton densities reported experimentally constrain the number of synapses on individual neurons, and ultimately in the whole microcircuit. These facts create interdependencies in the connectivity between different types of neuron. In other words, the number of synapses formed on one type of neuron constrains the synapses that can be formed on other types, creating a multi-constraint problem: the algorithm has to derive the numbers of connections and synapses between all types of neuron simultaneously—a Sudoku-like approach.

Here we describe the derivation and validation of the algorithm. The interdependencies described above, combined with additional insights into the properties of the microcircuit, make it feasible to predict the micro-scale connectome from sparse experimental data (also see Egger et al., 2014). A first draft predicted connectome using this algorithm is presented in Markram et al. (2015). The predicted connectome contains 7.8 million connections and 36 million synapses.

## Results

### A theoretical framework for microcircuit connectivity

We identified five fundamental anatomical properties of microcircuit connectivity and developed a theoretical framework that describes their interdependencies and facilitates simultaneous derivation of the connectivity between all neurons. The first property is the neuron density in each layer and for each morphological type (*C*_*d*_). An increase in the density of a certain type of neuron potentially affects the connectivity of the whole microcircuit. Increased density implies increased neuron numbers. Thus, maintaining the same level of connectivity *to* neurons of that type requires more axons and/or higher bouton densities, and maintaining the same connectivity *from* neurons of that type implies an increase in the density of the synapses they form on postsynaptic targets. This would in turn reduce the space available for extrinsic synapses formed by afferent axons from outside the microcircuit (i.e., it would increase the fraction of synapses between the neurons; intrinsic synapses). The second fundamental property is the total length of the axons formed by neurons of specific types, which connect to other neurons within the microcircuit (*A*_*l*_)—a key factor in determining the number of appositions and synapses that can be formed. The third fundamental property is the density of boutons (*B*_*d*_) on the axons of each type of neuron, which determines how many synapses can form for a given *A*_*l*_. Together with *A*_*l*_ and the number of synapses per connection, *B*_*d*_ also determines the range for the total number of connections that a neuron can form and creates interdependencies between all neuron types. For example, with constant *B*_*d*_, any increase in connectivity to one type of neuron must be compensated by a reduction in the number of connections to other types. The fourth fundamental property is the mean number of synapses per connection (*S*_*m*_) for each connection type (connections between pairs of neurons belonging to specified morphological types). This scales the number of target neurons that can be contacted by a presynaptic neuron with a given number of boutons. An important related property is the standard deviation of the number of synapses per connection (*S*_*sd*_), which reflects the biological variability of anatomical strength of connections. The fifth property is the connection probability for each type of connection (*C*_*p*_). Together with *C*_*d*_, this property determines the total number of connections formed by a neuron and the *S*_*m*_ required to reach the correct *B*_*d*_.

**Figure 1:**
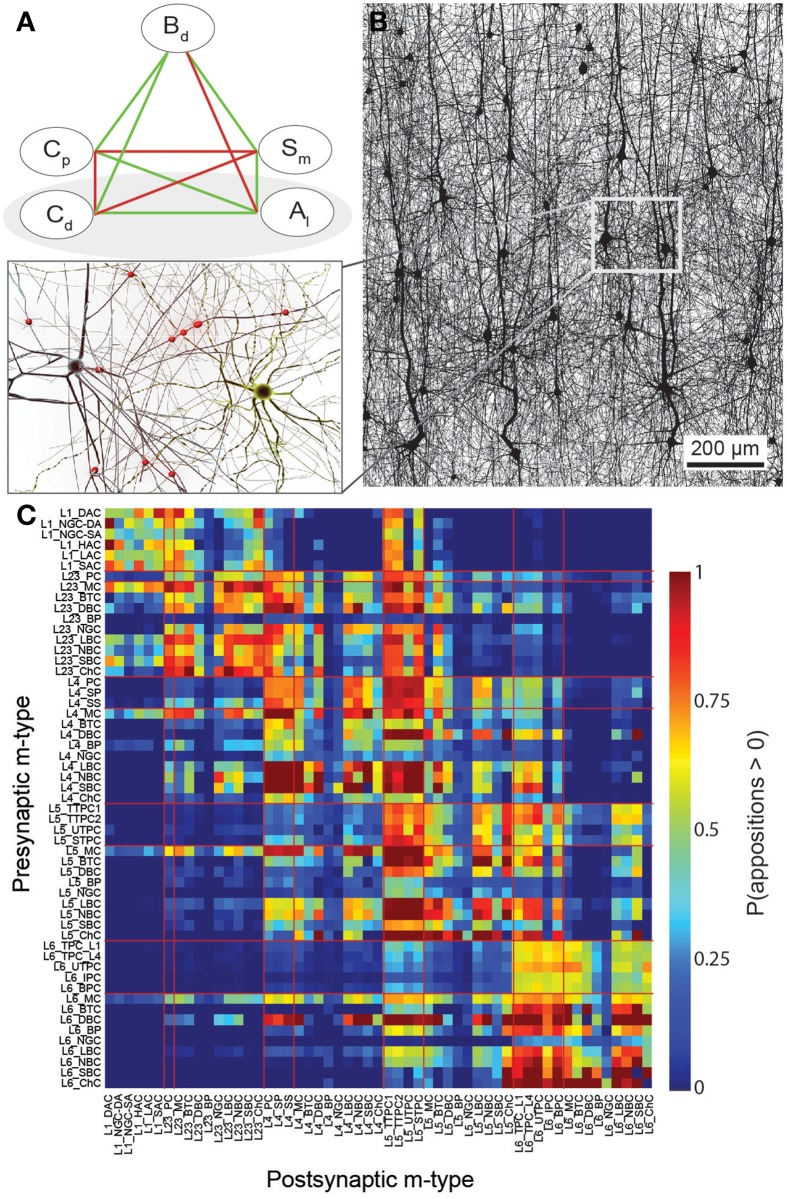
**A connectome selected from incidental appositions is constrained by connectivity measures and circuit parameters**. **(A)** Logical dependencies between connectivity metrics in a pathway. Green edges indicate that when one metric increases, the other also increases, provided the rest remains unchanged, vice versa for red. Metrics are: Bouton density (*B*_*d*_), connection probability (*C*_*p*_), mean number of synapses per connection (*S*_*m*_), cell density (*C*_*d*_) and axonal length (*A*_*l*_). **(B)** Part of the unitary microcircuit after all morphologies are placed (5% cell density shown). The resulting high density of fibers leads to a myriad of pairwise morphological appositions. Magnification: Example of a pair of morphologies with all 12 axonal appositions between them highlighted. **(C)** Resulting connection probabilities for neuron pairs within 100 μm of each other (horizontal distance between somas) in all types of pathways, if a synapse was placed at every single apposition.

The dependencies are illustrated qualitatively in Figure 1A. If we define ${\hat{C}}_{d}$ as the integral of neuron density (*C*_*d*_) over the spatial extent of the axonal arborization of a presynaptic neuron type (i.e., total number of potential target neurons), and ${\hat{C}}_{p}$ as the mean of the soma-distance-dependent connection probability (*C*_*p*_) over the same extent, then the dependencies between the properties are expressed by:

(I) $$\begin{array}{l} \underset{b\in M}{\sum }S_{m}\langle a,b\rangle \cdot {Ĉ}_{p}\langle a,b\rangle \cdot {Ĉ}_{d}\langle b\rangle =A_{l}\langle a\rangle \cdot B_{d}\langle a\rangle , \end{array}$$

where 〈*a, b*〉 refer to the pre and postsynaptic morphological types, and *M* to the set of all types in the reconstruction.

Since the neuronal composition is given, the values for *C*_*d*_ and *A*_*l*_ are fixed (Markram et al., 2015). Previous experimental studies provide sparse data for the remaining three microcircuit properties (*B*_*d*_, *S*_*m*_, *C*_*p*_). Combined with the principles just described, the interdependencies between these properties make it theoretically feasible to use sparse data for a few types of connections to constrain the solution for all types. Thus:

(II) $$\begin{array}{r} \underset{b\in U}{\sum }S_{m}\langle a,\;b\rangle \cdot {Ĉ}_{p}\langle a,\;b\rangle \cdot {Ĉ}_{d}\langle b\rangle \\ =A_{l}\langle a\rangle \cdot B_{d}\langle a\rangle -\underset{b^{*}\in K}{\sum }S_{m}\langle a,\;b^{*}\rangle \cdot {Ĉ}_{p}\langle a,\;b^{*}\rangle \cdot {Ĉ}_{d}\langle b^{*}\rangle , \end{array}$$

where the set of all morphological types is separated into one set, whose properties are unknown (*b* ∈ *U*), and a second set where they are known (b^*^ ∈ *K*). However, this formulation on its own provides predictions only for the sum of the product of all unknown *S*_*m*_ and *Ĉ*_*p*_. Assuming no specificity for any particular *b* ∈ *U*, Peters' rule can be used to split the sum into predictions of the $S_{m}\cdot {\hat{C}}_{p}$ product for individual *b* ∈ *U*. However, predicting *S*_*m*_ and *Ĉ*_*p*_ separately requires further information (see below).

### Established principles of connectivity

In a neocortical microcircuit, the arbors of the majority of neurons overlap, coming into close contact with most other neurons, and providing nearly all-to-all potential connectivity, at least within a given layer (Kalisman et al., 2005). We refer to points of contact between neurons as *appositions*, defined as contacts where the distance (a touch distance) between the neurons is less that the maximum distance (see also Hill et al., 2012) that can be bridged by a spine on the postsynaptic neuron, the swelling of a bouton on the axon, and minor bending of the axon (i.e., no directed axonal growth is required to form the contact (Silver et al., 2003; Karube et al., 2004; Kawaguchi, 2005, for reviews see Somogyi et al., 1998; Nimchinsky et al., 2002; Stepanyants and Chklovskii, 2005; Sala and Segal, 2014). A previously developed supercomputer-based application (Kozloski et al., 2008) was used to identify all appositions in the digitally reconstructed microcircuit (Figure 1B). Defining a maximal touch distance of 2.5 μm for excitatory and 0.5 μm for inhibitory synapses, we found ~600 million appositions, and the same, nearly all-to-all potential connectivity within a layer observed in experiments (Figure 1C). The first rule of connectivity is, therefore, that virtually all neurons within a layer of a microcircuit are potentially connected—*a tabula rasa rule.* The next step for the algorithm is to identify a subset of these appositions that can form biologically viable synaptic connections.

Biologically, synapses can only form at appositions. However, in the reconstruction, digitally reconstructed neurons are placed randomly in the same 3D volume, and it was not clear whether this procedure could accurately reproduce synapse locations in biological tissue. A recent study resolved this issue, demonstrating that, provided the vertical orientations and layer placement of neurons are respected, this procedure does indeed reproduce the statistical distributions of synapse locations observed in biological studies (Hill et al., 2012), and that synapse locations on dendrites are invariant with respect to the specific exemplar morphologies used. The second rule thus states that the location of synapses is established by the incidental appositions of semi-randomly placed neurons—*the synapse location rule*. This rule implies that the algorithm does not need to specifically align neurons to reproduce the biological locations of synapses. There are two important exceptions: (1) excitatory axons never form synapses on excitatory somata; (2) only Chandelier axons form synapses on axons of other neurons (Somogyi et al., 1982, 1998; DeFelipe, 1999; Szabadics et al., 2006). The algorithm implements these exceptions by prohibiting the formation of excitatory synapses on excitatory somata, and by allowing Chandelier axons to form synapses exclusively on the axonal initial segments of pyramidal neurons.

Given these two rules, one approach would be to derive the connectivity based purely on appositions. However, when we did so, we found that the predicted density of synapses on axons was considerably higher (> 3 μ*m*^−1^ for most m-types, **Table 2**) than reported bouton densities (≈ 0.2μ*m*^−1^, Wang et al., 2002; Romand et al., 2011). In fact, converting all appositions to synapses would lead to densities approximately 18 times higher than the value observed in biology. Thus, conversion of all appositions to synapses would violate Equation (I). Earlier studies had also observed that a connectome, in which all axo-dendritic appositions become synapses, would be massively over-connected and proposed that, while each apposition is a *potential synapse*, actual synapses form at only a fraction of them—the *filling fraction* (Stepanyants et al., 2002; Stepanyants and Chklovskii, 2005). We therefore use this finding as the third rule—*the fractional conversion rule*.

### Simple apposition pruning cannot account for synapse numbers

**Figure 2:**
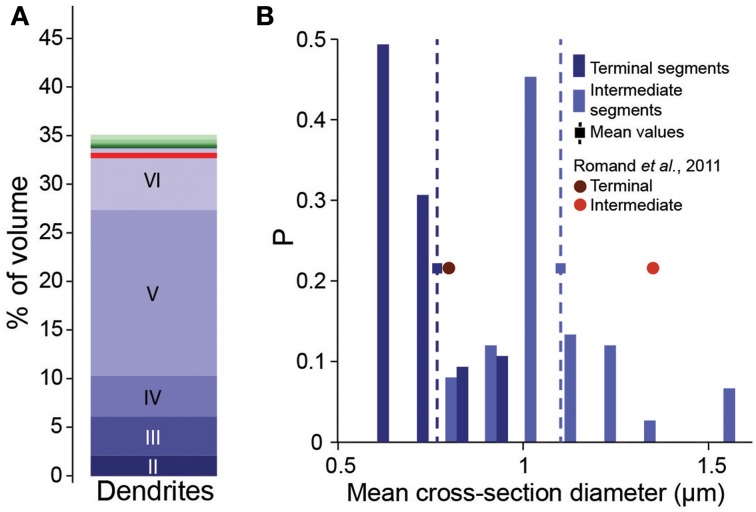
**Validation of volumetric dendrite densities. (A)** Fraction of the volume occupied by dendrites in a reconstructed microcircuit, surrounded by neighboring microcircuits on six sides. Contributions from cells residing in different layers are indicated by different shades of blue. Contributions from the surrounding microcircuits are stacked in different shades of green. Red solid horizontal lines indicate biological volume fractions in hippocampus (Mishchenko et al., 2010). **(B)** Distribution of diameters of basal dendrites of L5_TTPCs. Blue bars: reconstruction for (dark) terminal and (light) intermediate segments, squares and dashed lines indicate the mean; red circles: mean values for P14 of Romand et al. (2011).

As a first attempt to implement the fractional conversion rule, we applied Peters' Rule, which has been used extensively to predict connectivity from morphology (Peters and Feldman, 1976; Peters et al., 1979; Braitenberg and Schüz, 1998; Braitenberg, 2001; Stepanyants and Chklovskii, 2005). This rule proposes that the actual number of synapses along a dendrite is a constant fraction of the number of potential synapses. The simplest way of implementing the rule would be to select a fraction of potential synapses randomly, converting each into an actual synapse with a constant, independent probability. We therefore set this probability to the estimated overall *B*_*d*_ (0.2μ*m*^−1^), divided by mean *B*_*d*_, based on potential synapses (4.7μ*m*^−1^). One can only expect to reach this full biological density, if axons are fully utilized to form synapses. In the reconstruction, this was only true if all postsynaptic targets were present (i.e., all dendrites and somas were fully represented). Even though the neuron densities were provided and validated in the accompanying study (Markram et al., 2015), we tested the prediction by comparing the volume of the neuropil occupied by dendrites in the reconstruction with the volume found in EM studies. Dendrites occupied 35% of the volume (Figure 2A), which compares reasonably well with the 33% reported previously in Hippocampus (Mishchenko et al., 2010). The validity of the volume comparison requires that the digitally reconstructed neurons accurately capture dendritic diameters. Comparison showed that the digitally reconstructed diameters provide a reasonable match to previous results and that previous estimates fell within the range of the results in the reconstruction (Romand et al., 2011; Figure 2B). Diameters of hippocampal dendrites measured in EM also matched the reconstruction (not shown, Mishchenko et al., 2010). We therefore conclude that using reported biological *B*_*d*_ in Equation I is a good first approximation.

**Figure 3:**
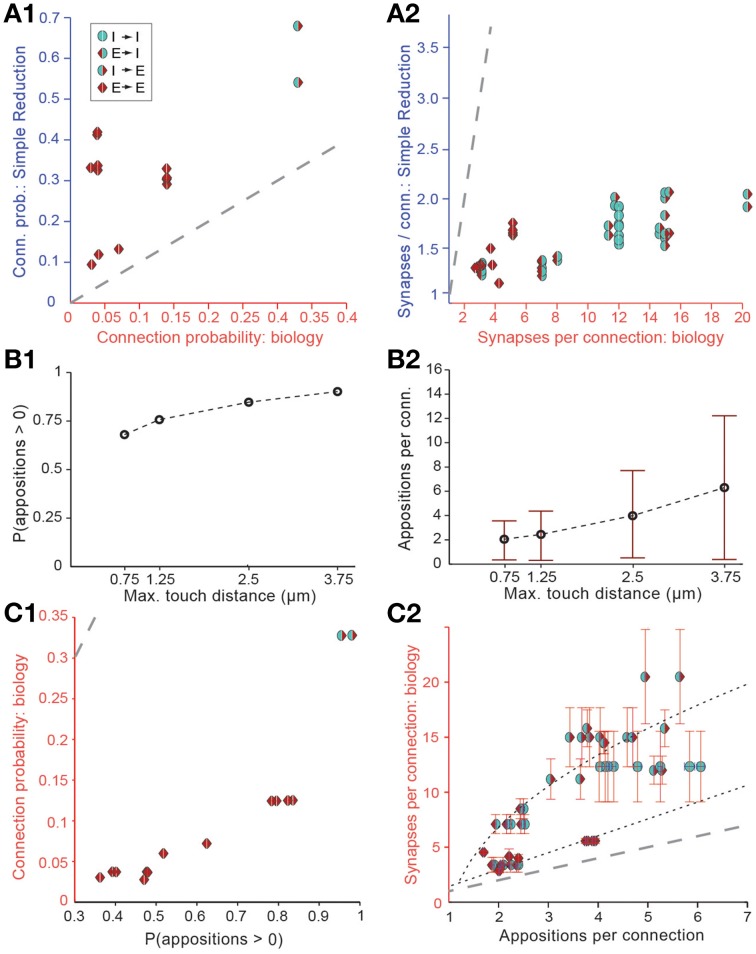
**Simple approaches to filtering of potential synapses do not reproduce biological connectivity**. **(A)** Comparing the results of a simple random pruning of potential synapses to biological data: Potential synapses were removed with a uniform, independent probability, otherwise an actual synapse is allowed to form. **(A1)** Resulting connection probabilities, **(A2)** resulting mean number of synapses per connection (maximal distance 100 μm). Markers indicate the type of pathway, red triangle: excitatory, blue semicircle: inhibitory. Left side indicates the type of the presynaptic m-type, right side indicates the postsynaptic m-type. Gray, dashed line indicates the identity (*x* = *y*). **(B)** Connection probability **(B1)** and synapse numbers **(B2)** in a network of L5_TTPC2 neurons (maximal distance 100 μm) based on potential synapses when the maximal reach of potential synapses is changed. Bars indicate means, error bars standard deviations. **(C)** Comparing connectivity based on all potential synapses to biological data. **(C1)** Resulting connection probabilities, and **(C2)** mean number of potential synapses per connection. Markers and gray line as in **(A)**. Black, dashed lines indicate the fits used to predict the mean number of synapses.

Randomly removing a fraction of potential synapses (i.e., of appositions) in this manner reduced the density of potential synapses to biological levels, but also reduced *C*_*p*_ and *S*_*m*_ (Figures 3A1,A2). This produced a unimodal distribution for the numbers of potential synapses between pairs of neurons, in which most connections only had one potential synapse (see also Hellwig, 2000). Such a distribution contradicts experimental findings, which show that the distribution of synapse numbers is bimodal, i.e. that about 90% of neuron pairs are not connected (see also Bienenstock et al., 1982), and that the remaining pairs are always connected by multiple synapses (Deuchars et al., 1994; Markam et al., 1997; Markram et al., 2004; Feldmeyer et al., 1999, 2006; Reyes and Sakmann, 1999; Wang et al., 2002; Silver et al., 2003; Silberberg and Markram, 2007; Frick et al., 2008). Therefore, the fourth rule states that connections always involve multiple synapses—the *multi-synapse rule*. To enforce this rule, the algorithm prunes connections with too few potential synapses (see below).

We also attempted to reach biological *B*_*d*_ values by enforcing stricter rules with respect to what constitutes a potential synapse, i.e., by reducing the maximal reach of a potential synapse, its *touch distance*, toward 0 μm. This procedure reduced the number of potential synapses to a level compatible with biological bouton densities, but also led to significant changes in *C*_*p*_ (Figure 3B1) and *S*_*m*_ (Figure 3B2), with lower touch distances leading to a small decrease in *C*_*p*_ and a large decrease in *S*_*m*_. In brief, it failed to reproduce biological connectivity, which is characterized by low *C*_*p*_ and high *S*_*m*_.

The simple implementation of Peters' Rule and the reduction in the touch distance both led to results that violated the multi-synapse rule. This indicates that neither is a valid solution for pruning appositions. Although both approaches produce valid solutions for Equations (I) and (II), and both yield correct values for the product *S*_*m*_ · *Ĉ*_*p*_, in both cases, *S*_*m*_ itself is too low.

### General, multi-synaptic, and plasticity-reserve pruning

Fares and Stepanyants have proposed a two-step process which implements the fractional conversion rule while maintaining the multi-synapse rule (Fares and Stepanyants, 2009). The first step is similar to the simple implementation of Peters' Rule described above, again starting with a potential synapse at each apposition and randomly removing a fraction of them; the second selectively removes all connections with a low number of potential synapses. However, this approach cannot determine the mean number of synapses for unknown connection types, and cannot, therefore, be generalized to the whole microcircuit. It also does not use *B*_*d*_ or *C*_*p*_ as constraints. This means it cannot exploit interdependencies in the parameters to constrain the solution.

Although our estimate of *C*_*p*_ based on all potential synapses was approximately five times higher than the biological *C*_*p*_, we nonetheless found a strong correlation between these probabilities and previously reported connection probabilities for different types of connection (Figure 3C1; *r* = 0.88, *p* < 0.01, *N* = 13). Similarly, while *S*_*m*_ based on all potential synapses was consistently much lower than in experiments, the number of potential synapses and the number of reported synapses per connection were also correlated (Figure 3C2; *p* < 0.01, *r* = 0.78, *N* = 38), with two distinct relationships, one for excitatory to excitatory connections, and one for all other connection types (Figure 3C2). Minimizing the sum of absolute errors, the optimal linear fit to the E→E data was $S_{m}=1.5\cdot S_{m}^{struc}$, where $S_{m}^{struc}$ is defined as the mean number of potential synapses per connection. For other types, we optimized a general square root function which yielded $S_{m}=9\cdot \sqrt{S_{m}^{struc}-1}-2$. This indicates that the numbers of potential synapses, at appositions between different types of neurons, carry information about the number of connections and synapses they form. We used this information to modify the approach of Fares and Stepanyants (2009) in a number of ways.

**Figure 4:**
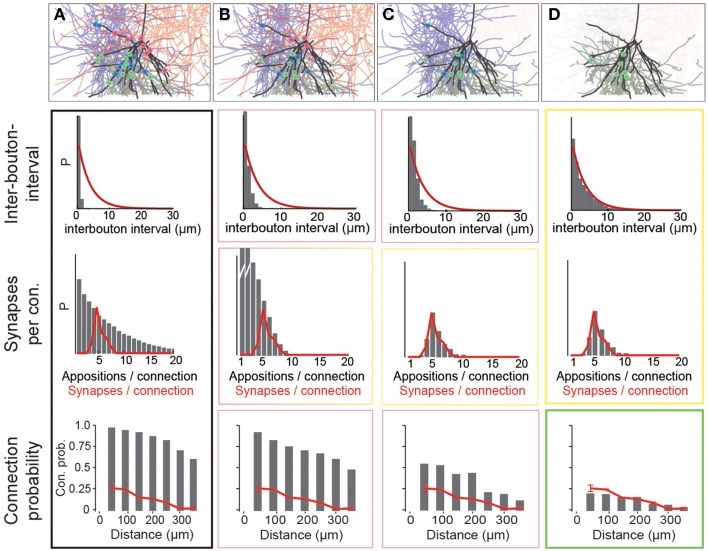
**Schematic of the three pruning steps**. For an exemplary pathway (L5_TTPC2 to L5_TTPC2), we show how the distributions of inter-bouton intervals (IBI, inverse of bouton density), synapses per connection and connection probability are matched after three pruning steps. **(A)** Connectivity based on all potential synapses (i.e., appositions) is characterized by short IBIs, an extremely wide distribution of potential synapses per connection, and almost 100% connection probability. Top: An exemplary L4_PC surrounded by 3 LBCs with all potential synapses highlighted. **(B)** Randomly removing a fraction (1−*f*_1_) of potential synapses removes the right hand side of the distribution of synapses per connection. Top: This removes a fraction of potential synapses in all three connections. **(C)** Removing connections formed by too few potential synapses also culls the left hand side, but inter-bouton intervals are still too short. Top: The panel shows the removal of one complete connection that does not have the required number of potential synapses. **(D)** The last step randomly removes more connections, leading to correct inter-bouton-intervals and connection probabilities only slightly below reported values emerge. Top: One of the two remaining connections is (randomly) removed.

Using data for the well-characterized connections formed between layer 5 thick-tufted pyramidal neurons (Markam et al., 1997) as a benchmark, we found that the distribution of the number of potential synapses per connection in the reconstructed microcircuit was much wider than the distribution of actual synapses per connection, observed experimentally (Figure 4A; potential synapses in gray, experimental values in red). In other words, the SD of the number of synapses per connection (*S*_*sd*_) was considerably larger. This was because the reconstruction displayed an excess, both of connections with too few potential synapses (left side of the distribution), and of connections with too many (right side). This was true for all connection types that have been characterized experimentally (not shown). Therefore, the first step in the algorithm randomly eliminated potential synapses until the right side of the distributions matched the biological data, where available, and predicted *S*_*sd*_ (see below) where they were not (Figure 4B). The first parameter of the algorithm—*f*_1_—is thus the fraction of potential synapses that remains in an m-type to m-type specific connection after *general pruning*. Based on the finding that the number of potential synapses per connection always follows a geometric distribution (*P*(*n* = *k*) = (1−*p*)^*k*−1^ · *p*, see Figure 4A; van Ooyen et al., 2014; but see Fares and Stepanyants, 2009), *f*_1_ can be calculated from *S*_*sd*_ as follows (for detailed derivation see Methods):

(IIIa) $$\begin{array}{l} p^{\prime }=\frac{1}{S_{sd}+0.5}, \end{array}$$

(IIIb) $$\begin{array}{l} f_{1}=\frac{p}{1-p}\cdot \frac{1-p^{\prime }}{p^{\prime }}. \end{array}$$

The second step pruned all potential synapses belonging to connections with too few potential synapses to match the left side of the biological distribution (Figure 4C, see Methods). The second parameter of the algorithm, μ_2_, therefore defines the placement of a sigmoidal cutoff function for *multi-synaptic pruning*, which can be calculated from *S*_*m*_ and *S*_*sd*_, using the expression (for detailed derivation see Methods):

(IV) $$\begin{array}{l} \mu_{2}=0.5+S_{m}-S_{sd}. \end{array}$$

With the correct values of *f*_1_ and μ_2_, both *S*_*m*_ and *S*_*sd*_ matched biological data, where available, and predicted values where they were not. However, the values for *B*_*d*_ were still approximately four times higher than the biological values, even after accounting for the fact that a fraction of boutons form multiple synapses (Bopp et al., 2014). Additionally, if all remaining potential synapses were converted to synapses, there would be no room for rewiring of the microcircuitry. This would contrast with experiments on pyramidal neurons which have found a near doubling of connection probabilities following stimulation (Chklovskii et al., 2004; Lamprecht and LeDoux, 2004; Le Be and Markram, 2006; Neves et al., 2008; Holtmaat and Svoboda, 2009; Wilbrecht et al., 2010). The same experiments suggest that at most half of the possible multi-synapse connections are functionally active, and that the rest serve as a reservoir for rewiring plasticity. The s*ynapse location rule* implies that rewiring has no effect on the distribution of synapse locations. The fifth rule therefore states that only a fraction of potential multi-synapse connections are functionally realized—*the plasticity reserve rule*. Guided by this finding, we added a third pruning step, in which we randomly removed a fraction of the potential multi-synapse connections found after the second step (for the L5_TTPC pathway, the fraction was **0.19**). The third parameter—*a*_3_–was therefore the fraction of potential multi-synapse connections retained after *plasticity-reserve pruning*. This can be calculated from m-type specific *B*_*d*_ values as shown below (for detailed derivation see Methods):

(V) $$\begin{array}{l} a_{3}=\frac{B_{d}}{B^{2}}, \end{array}$$

where *B*^2^ refers to the bouton density for a given m-type if all potential multi-synapse connections were retained.

After this step, the reconstruction not only matched reported biological *B*_*d*_, but also reported biological distance-dependent connection probabilities for pyramidal neurons (on average 85% of the reported biological level, Figure 4D; lowermost graph, Perin et al., 2011). The finding that over 50% of multi-synapse connections were held in reserve, is consistent with the doubling of *C*_*p*_ following stimulation of the microcircuit, reported for this type of connection, (Le Be and Markram, 2006).

### Validation and robustness of the algorithm

The algorithm does not directly use biological data to prune potential synapses. Instead, they are pruned using the three parameters just described, which capture the interdependencies between *B*_*d*_, *S*_*m*_, and *C*_*p*_ and can therefore be derived using variable amounts of biological data. The algorithm allows for the use of biological data to directly specify the three parameters for connection types where data is available (the *biological* parametrization approach), and predict the three parameters for each connection type where they are not (the *derived* parametrization approach).

Given the extremely large parameter space and number of appositions (~600 million), the computational cost of iterative parameter optimization would be prohibitive. However, calculating a set of parameters for each m-type specific connection type from unpruned appositions yields a unique solution for each connection type without iteration. Values for individual neuron to neuron connections can then be derived statistically from these solutions.

**Figure 5:**
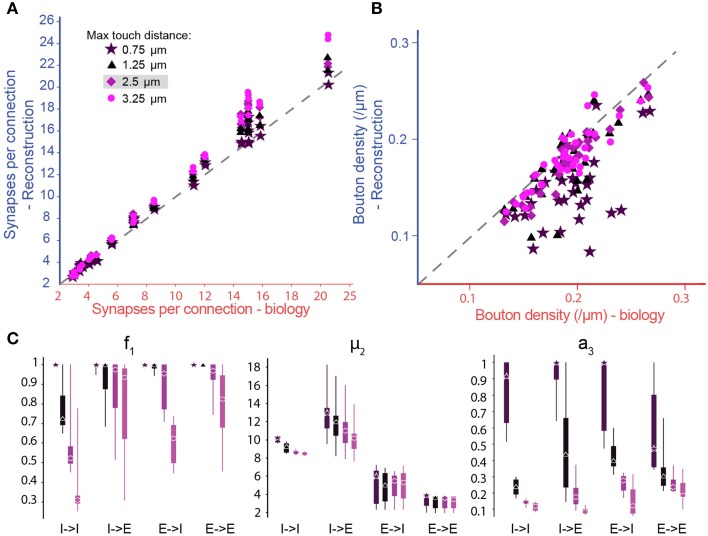
**Biological synapse numbers and bouton densities recreated for different touch distances**. **(A)** Resulting mean number of synapses per connection in pathways, where available biological data on mean and standard deviation of synapse numbers as well as bouton densities are used to fully constrain algorithm parameters. Results for different values of the maximal reach of potential synapses (touch distance): Stars: 0.75 μm; triangles: 1.25 μm; diamonds: 2.5 μm; circles: 3.75 μm. Mean values compared to biological values in Table 1. **(B)** Same for the bouton densities of individual m-types. Mean values compared to biological values in **Table 3**. **(C)** Box-plots of the parameter values determined by the biological parameterization procedure for inhibitory to excitatory (*I* → *E*), excitatory to inhibitory (*E* → *I*), inhibitory to inhibitory (*I* → *I*), and excitatory to excitatory (*E* → *E*) pathways and different touch distances (left to right). Markers indicate the median, thick lines the 25 and 75% percentiles and thin lines the full data spread.

The derivation of the parameters and the three step pruning process were validated against known biological data. A search of the literature for rat somatosensory cortex found only 38 m-type specific connection types where *B*_*d*_, *S*_*m*_, and *S*_*sd*_ have been measured experimentally, and 14 with estimates for *C*_*p*_ (see the accompanying paper, Markram et al., 2015). When we ran the algorithm on the whole microcircuit, we found a near perfect match between the number of synapses per connection in the reconstruction and the available biological data (Figure 5A; purple diamonds), and no statistical evidence for any mismatch (Table 1). We also observed no statistically significant differences between the bouton densities of the axons of presynaptic m-types and the available biological data (Figure 5B; purple diamonds; see Methods). We conclude, at this stage, that the algorithm can be applied to any connection type for which biological data is available. This validates the equations for the derivation of the parameters and the simultaneous derivation of connectivity for multiple connection types.

**Table 1:** **Mean number of appositions or synapses per connection for different touch distances**.

**Pathway**		**All**	**Pruned**								**Predictive**		**Bio**
**From**	**To**	**2.5 μm**	**0.75 μm**	***P***	**1.5 μm**	***P***	**2.5 μm**	***P***	**3.75 μm**	***P***	**2.5 μm**	***P***	
L23_BTC	L23_PC	3.43	15.0	0.50	15.9	0.27	16.6	0.21	17.4	0.20	13.3	0.38	15.0^6^
L23_LBC		4.12	15.0	0.39	16.3	0.25	16.7	0.25	17.3	0.25	16.0	0.64	14.5^6^
L23_MC		3.05	11.2	0.48	11.7	0.31	12.1	0.24	12.7	0.21	11.5	0.81	11.2^11^
L23_NBC		3.78	15.6	0.45	16.6	0.32	17.3	0.25	18.7	0.20	14.4	0.50	15.8^6^^,^^13^
L23_SBC		4.95	20.2	0.43	21.6	0.31	22.8	0.21	24.8	0.17	18.3	0.44	20.5^6^
L23_PC	L23_PC	2.01	2.8	0.45	2.8	0.47	2.9	0.49	2.8	0.44	3.1	0.63	2.9^1^
	L23_LBC	2.16	7.8	0.15	7.9	0.14	8.5	0.08	8.3	0.15	8.5	0.10	7.1^6^
	L23_NBC	2.09	3.8	0.27	3.8	0.28	3.9	0.26	3.7	0.32	8.0	0.00	3.4^6^^,^^13^
L23_LBC	L23_LBC	4.20	13.7	0.28	15.0	0.23	14.8	0.54	15.6	0.24	17.6	0.25	12.3^12^
L23_SBC		5.25	14.4	0.24	15.2	0.24	14.9	0.53	15.6	0.24	20.3	0.12	12.3^12^
L23_LBC	L23_SBC	4.02	13.2	0.34	14.4	0.25	15.0	0.55	15.7	0.26	16.0	0.35	12.3^12^
L23_SBC		4.79	13.8	0.26	14.9	0.23	15.4	0.49	15.7	0.23	18.9	0.16	12.3^12^
L4_BTC	L4_PC	3.67	15.1	0.46	16.3	0.23	16.6	0.45	18.1	0.17	14.8	0.91	15.0^6^
L4_LBC		4.12	15.1	0.37	16.0	0.28	16.2	0.58	16.9	0.27	15.0	0.87	14.5^6^
L4_MC		3.64	11.4	0.40	11.9	0.28	12.3	0.53	12.4	0.24	14.4	0.06	11.2^6^^,^
L4_NBC		5.34	16.5	0.33	17.5	0.25	18.0	0.50	18.5	0.22	19.0	0.35	15.8^6^^,^^13^
L4_SBC		5.65	21.4	0.33	22.7	0.23	23.9	0.40	24.4	0.21	19.7	0.83	20.5^6^
L4_PC	L4_LBC	2.24	7.7	0.20	7.9	0.16	7.9	0.34	7.9	0.20	8.3	0.15	7.1^6^
	L4_NBC	2.24	3.9	0.25	3.8	0.31	3.8	0.59	3.7	0.33	8.3	0.00	3.4^6^^,^^13^
L4_LBC	L4_LBC	4.31	13.9	0.28	14.0	0.29	14.1	0.61	14.6	0.29	15.2	0.36	12.3^12^
L4_SBC		6.06	14.1	0.29	14.5	0.29	14.8	0.54	14.6	0.27	20.8	0.13	12.3^12^
L4_LBC	L4_SBC	4.14	13.4	0.31	13.6	0.31	14.7	0.53	14.3	0.29	16.3	0.38	12.3^12^
L4_SBC		5.84	13.7	0.32	14.0	0.32	13.6	0.71	14.4	0.30	20.2	0.11	12.3^12^
L4_SP	L4_SP	2.05	3.4	0.49	3.6	0.32	3.5	0.68	3.5	0.35	3.1	0.44	3.4^2^^,^^8^
L4_SS	L23_PC	1.70	4.3	0.17	4.6	0.43	4.8	0.57	4.7	0.35	2.5	0.00	4.5^3^^,^^^10^^
L5_BTC	L5_TTPC1	4.59	17.3	0.16	17.6	0.15	18.4	0.28	18.9	0.13	18.3	0.28	15.0^6^
L5_MC		5.12	13.0	0.31	13.4	0.30	13.5	0.59	13.6	0.28	18.4	0.06	12.0^7^
L5_BTC	L5_TTPC2	4.70	16.8	0.17	17.9	0.13	18.8	0.25	19.3	0.12	18.9	0.24	15.0^6^
L5_MC		5.29	13.2	0.29	13.7	0.29	13.7	0.56	13.8	0.27	19.4	0.06	12.0^7^
L5_STPC	L5_STPC	2.40	4.0	0.46	4.2	0.32	4.2	0.71	4.3	0.26	3.9	0.77	4.0^4^
L5_TTPC1	L5_MC	2.43	9.0	0.34	9.1	0.34	9.2	0.64	9.5	0.31	9.4	0.58	8.5^7^
	L5_TTPC1	3.79	5.9	0.23	6.1	0.18	6.2	0.33	6.3	0.13	6.0	0.47	5.6^5^^,^^9^
	L5_TTPC2	3.75	5.9	0.22	6.2	0.16	6.1	0.35	6.3	0.13	5.9	0.57	5.6^5^^,^^9^
L5_TTPC2	L5_MC	2.50	9.2	0.31	9.2	0.31	9.2	0.66	9.7	0.30	9.7	0.47	8.5^7^
	L5_TTPC1	3.94	5.8	0.31	6.0	0.22	6.0	0.44	6.2	0.16	6.3	0.21	5.6^5^^,^^9^
	L5_TTPC2	3.89	5.9	0.28	6.0	0.22	6.0	0.45	6.1	0.17	6.2	0.27	5.6^5^^,^^9^
L6_BTC	L6_TPC_L1	4.04	16.1	0.23	17.5	0.13	18.3	0.26	19.5	0.13	16.9	0.51	15.0^6^
	L6_TPC_L4	3.83	15.9	0.26	17.1	0.14	18.2	0.24	19.3	0.14	16.0	0.69	15.0^6^

Mean number of potential synapses per connection in experimentally characterized m-type to m-type connections, based on all appositions (“All”) and after application of the pruning algorithm (“Pruned”) for different maximal touch distances. P-values of a t-test comparing the number of synapses of individual connections reported in the literature against samples in the reconstructed micro-scale connectome, are provided (low values indicate significant difference). Values < 0.05 are indicated in yellow, < 0.01 in orange, < 0.001 in red.

The column “Bio” is based on results from:

1 Feldmeyer et al., 2006;

2 Feldmeyer et al., 1999;

3 Feldmeyer et al., 2002;

4 Le Bé et al., 2007;

5 Markam et al., 1997;

6 Markram et al., 2004;

7 Silberberg and Markram, 2007;

8 Petersen and Sakmann, 2000;

9 Romand et al., 2011;

10 Lübke, 2003;

11 Fino and Yuste, 2011;

12 Gupta et al., 2000;

13 Wang et al., 2002.

To explore the robustness of the algorithm, we tested its sensitivity to changes in the touch distances used to define potential synapses (Figure 5A, different colors). By changing the touch distance from 0.75 to 3.25 μm, we found that the resulting numbers of synapses per connection, after pruning, were less sensitive to changes in the touch distance than the numbers of potential synapses per connection that went into the algorithm. This was because the algorithm could maintain the number of synapses per connection by adjusting parameters, increasing *f*_1_ (i.e., less general pruning) and/or increasing μ_2_ (i.e., more multi-synapse pruning). Furthermore, the algorithm could still achieve biological *B*_*d*_ by increasing *a*_3_, although if *a*_3_ became too large, this could lead to violation of the *plasticity reserve rule*. With many connection types, it was impossible to fully reproduce the values of the biological properties using a maximal touch distance of 0.75 μm and, for a few, it was still impossible with a distance of 1.25 μm (Figure 5B, Tables 1, 2). These failures were due to the lack of sufficient potential synapses to solve the multi-constraint problem. This made it necessary to assign different priorities to the properties the algorithm had to reproduce. For the data in Figure 5, we gave the highest priority to *S*_*m*_, then *S*_*sd*_, with *B*_*d*_ last. Consequently, reproduction of biological values failed in the inverse order. When the algorithm failed to reproduce biological values for a property, the relevant parameter reached its maximum allowed value (Figures 5B,C, right). Thus, a value of 1.0 for *a*_3_ indicates that reaching biological bouton density would require all available multi-synapse connections, or even more. However, the *plasticity reserve rule* states that **a**_3_ < < **1**. When the maximal touch distance used to define potential synapses was set to 0.75 μm, 17 connection types violated this condition. At 1.25 μm, only two violations were observed. At distances of 2.5 μm and above, all 38 tested connection types satisfied the condition. This finding suggests that the required optimal touch distance for defining potential synapses is ~2.5 μm.

**Table 2:** **Connection probabilities for distances up to 100 μm**.

**Pathway**		**All appositions**				**Pruned**		**Bio**
**From**	**To**	**0.75 μm**	**1.5 μm**	**2.5 μm**	**3.75 μm**	**2.5 μm**	***P***	
L23_PC	L23_PC	0.44	0.50	0.55	0.59	0.06	0.43	0.055^8^
L4_SS	L23_PC	0.25	0.32	0.36	0.40	0.01	0.06	0.03^1^^,^^7^
L4_SP	L4_SP	0.52	0.57	0.62	0.66	0.06	0.41	0.07^5^
L5_MC	L5_TTPC1	0.89	0.95	0.98	0.99	0.10	0.0	0.33^4^
	L5_TTPC2	0.88	0.94	0.97	0.99	0.10	0.0	0.33^4^
L5_STPC	L5_STPC	0.36	0.42	0.47	0.52	0.05	0.08	0.03^2^
L5_TTPC1	L5_TTPC1	0.62	0.71	0.78	0.85	0.07	0.0	0.12^3^
L5_TTPC2		0.64	0.72	0.79	0.85	0.09	0.01	0.12^3^
L5_TTPC1	L5_TTPC2	0.66	0.74	0.82	0.88	0.08	0.0	0.12^3^
L5_TTPC2		0.66	0.75	0.83	0.9	0.09	0.01	0.12^3^
L6_TPC_L1	L6_TPC_L1	0.37	0.44	0.48	0.52	0.07	0.39	0.039^6^
L6_TPC_L4		0.37	0.43	0.48	0.53	0.06	0.43	0.039^6^
L6_TPC_L1	L6_TPC_L4	0.33	0.37	0.40	0.42	0.08	0.38	0.039^6^
L6_TPC_L4		0.34	0.37	0.39	0.42	0.05	0.44	0.039^6^

Mean connection probabilities in experimentally characterized m-type to m-type connections, based on all potential synapses (“All appositions”) and after application of the pruning algorithm (“Pruned”) for different maximal touch distances. P-values as in Table 1. The column “Bio” is based on results from:

1 Feldmeyer et al., 2002;

2 Le Bé et al., 2007;

3 Perin et al., 2011;

4 Silberberg and Markram, 2007;

5 Petersen and Sakmann, 2000;

6 Beierlein and Connors, 2002;

7 Lübke, 2003;

8 Holmgren et al., 2003.

In the case of *f*_1_, we observed very wide distributions of values between major classes of connection (I–I—*inhibitory to inhibitory*, I–E—*inhibitory to excitatory*, E–I—*excitatory to inhibitory*, E–E—*excitatory to excitatory*; Figure 5C, left) and even within classes. In contrast, μ_2_ values were tightly distributed, and only varied slightly with touch distance (Figure 5C, central panel). With moderate reductions in touch distance, the algorithm can solve the multi-constraint problem by applying less pruning (i.e., by increasing *f*_1_). Only when *f*_1_ had already reached its maximal value of 1, was it necessary to increase multi-synapse pruning (i.e., to increase μ_2_). This led to estimates for the minimal number of synapses needed to stabilize a connection in a given connection class. The estimate was significantly higher for inhibitory (12.52 for I–I, 13.82 for I–E) than for excitatory connections (6.56 for E–I, 3.50 for E–E), a finding that matches the experimental data (for example compare Markam et al., 1997; Markram et al., 2004; Silberberg and Markram, 2007). Together, these results show that it is impossible to use a single set of parameters for all connection types, although it may be possible for types within one of the major classes. Fares and Stepanyants found that a single set of parameters leads to good matches for the three E–E connection types they studied. These findings match our own. On the other hand, Shepherd et al. (2005) found that the ratio of the functional to “geometrical connection strength” (i.e., axo-dendritic overlap) for a number of trans-laminar E–E connection types depended on the layers involved and even on the position of cells in a barrel. This seems to indicate that different sets of parameters might be needed to relate structure to function in these cases. However, the functional connection strength they measured also depended on the strength of individual synapses (synaptic weight), and was not a measure of synapse numbers alone.

### Validity of the predicted connectivity

To test the possibility of predicting microcircuit properties purely from appositions, we used predicted values for each of the microcircuit properties (the *derived* parametrization approach), without using connection specific biological data. For *S*_*m*_ we relied on the relationship between potential synapses (i.e., appositions) per connection and synapses per connection, as shown in Figure 3C. While Equation II alone could only yield a result for the product *S*_*m*_ · *Ĉ*_*p*_, this new finding makes it possible to separately predict the two values. We also relied on the finding that the distribution of synapses per connection was typically narrow (mean c.v. of 0.32 for available biological data). We could then use the coefficient of variation for the biological data, combined with the predicted *S*_*m*_, to predict *S*_*sd*_. For *B*_*d*_ we used an average value (0.2μ*m*^−1^, see Table 2; Methods). Although distance has a profound effect on the value of the *C*_*p*_, relatively few studies normalize their estimates with respect to this parameter (Markam et al., 1997; Holmgren et al., 2003), and fewer still provide a distance-dependent profile (Perin et al., 2011). This means that the available biological data for *C*_*p*_ is largely unusable for this algorithm. We therefore decided that the algorithm should not rely on biological estimates of *C*_*p*_. Instead we predicted *C*_*p*_ using the predicted value of *S*_*m*_ (see Equation I).

**Figure 6:**
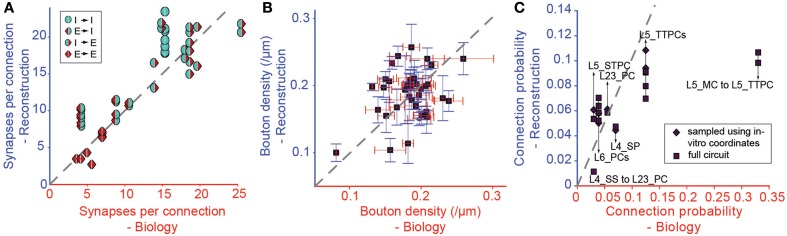
**Validation of the Predicted Connectivity**. Results emerging from the biological parametrization: **(A)** Comparison of the resulting mean number of synapses per connection to biology. Markers indicate the type of pathway as in Figure 3. **(B)** Comparison of bouton densities of a number of m-types to biology. Purple squares: mean densities, red error bar: standard error of mean (SEM) of biological data, blue error bar: SEM of model data. **(C)** Comparison of the mean connection probabilities in the model against biological data. Squares indicate connection probabilities of all cells within 100 μm, diamonds connection probabilities resulting from an *in silico* patch experiment (see Methods).

We tested the derived parametrization for connection types with previously reported values for *S*_*m*_ (Figure 6A), finding a highly significant match (*r* = 0.87, *p* < 0.01, *N* = 38). This is an indication that it is possible to accurately and simultaneously predict *S*_*m*_ for multiple connection types. Only three of the 38 connection types (see Table 1) displayed statistically significant differences (*p* < 0.05) between predicted and biological values. Synapse numbers were overestimated for excitatory connection types onto one type of basket cell and underestimated for the connection from L4 spiny stellate cells onto L2/3 pyramidal cells (see Table 1).

In this approach, we replaced connection type specific bouton densities with the overall mean bouton density (0.2μ*m*^−1^). Under these conditions, mean values for individual m-types correlated poorly with known biological values (Figure 6B, *r* = 0.38, *p* = 0.016, *N* = 40), but around half of the individual m-types showed no statistically significant difference between the predicted and the biological bouton densities for individual neurons (Table 3). These results imply that a single value for the *B*_*d*_ of all m-types is insufficient to fully constrain individual *B*_*d*_ for each m-type. However, because of the interdependencies between the solutions for each connection, it may not be necessary to measure the *B*_*d*_ for every m-type.

**Table 3:** **Mean densities of appositions and bouton densities after pruning for different touch distances**.

**m-Type**	**All**	**Pruned**								**Predictive**		**Bio**
	**2.5 μm**	**0.75 μm**	***P***	**1.5 μm**	***P***	**2.5 μm**	***P***	**3.75 μm**	***P***	**2.5 μm**	**P**	
L1_DAC	3.32	0.13	0.12	0.13	0.12	0.13	0.07	0.13	0.11	0.15	0.37	0.18
L1_DLAC	3.53	0.18	0.73	0.19	0.91	0.20	0.97	0.20	0.94	0.23	0.31	0.20
L1_HAC	2.96	0.16	0.01	0.20	0.86	0.19	0.45	0.21	0.95	0.19	0.31	0.21
L1_NGC_DA	3.28	0.16	0.33	0.17	0.52	0.16	0.28	0.18	0.61	0.19	0.87	0.20
L1_NGC_SA	3.25	0.16	0.44	0.16	0.47	0.16	0.62	0.16	0.45	0.18	0.89	0.18
L1_SLAC	3.51	0.13	0.23	0.12	0.18	0.15	0.53	0.13	0.17	0.15	0.49	0.18
L23_BP	3.27	0.20	0.83	0.20	0.81	0.19	0.89	0.20	0.86	0.23	0.56	0.19
L23_BTC	2.82	0.14	0.00	0.19	0.71	0.20	0.86	0.20	0.88	0.18	0.23	0.20
L23_DBC	3.29	0.24	0.17	0.25	0.48	0.24	0.20	0.25	0.50	0.20	0.00	0.27
L23_LBC	3.20	0.10	0.00	0.13	0.02	0.14	0.04	0.15	0.07	0.17	0.97	0.17
L23_MC	3.01	0.14	0.00	0.23	0.59	0.23	0.70	0.24	0.98	0.19	0.01	0.24
L23_NBC	2.99	0.13	0.00	0.20	0.71	0.21	0.97	0.21	0.99	0.19	0.42	0.21
L23_SBC	3.26	0.14	0.00	0.21	0.54	0.21	0.32	0.21	0.47	0.20	0.15	0.23
L4_BP	3.34	0.12	0.67	0.11	0.63	0.11	0.59	0.11	0.60	0.18	0.45	0.13
L4_BTC	3.25	0.15	0.12	0.18	0.88	0.17	0.53	0.19	0.90	0.21	0.37	0.19
L4_DBC	3.46	0.26	0.95	0.27	0.89	0.27	0.89	0.26	0.91	0.22	0.26	0.26
L4_LBC	3.60	0.15	0.00	0.17	0.09	0.19	0.28	0.18	0.15	0.20	0.62	0.21
L4_MC	3.22	0.16	0.02	0.17	0.22	0.19	0.59	0.19	0.39	0.18	0.38	0.20
L4_NBC	3.53	0.18	0.38	0.20	0.76	0.19	0.73	0.20	0.66	0.24	0.01	0.19
L4_SBC	3.69	0.15	0.05	0.18	0.50	0.19	0.82	0.19	0.60	0.22	0.55	0.20
L4_SP	3.89	0.18	0.00	0.21	0.46	0.21	0.50	0.21	0.66	0.24	0.00	0.22
L4_SS	3.42	0.14	0.00	0.17	0.04	0.17	0.08	0.17	0.33	0.20	0.01	0.18
L5_BP	3.76	0.16	0.94	0.16	0.91	0.16	0.86	0.16	0.91	0.24	0.02	0.16
L5_BTC	3.93	0.13	0.23	0.12	0.23	0.13	0.27	0.13	0.30	0.19	0.28	0.16
L5_DBC	3.84	0.19	0.46	0.19	0.57	0.19	0.58	0.20	0.73	0.22	0.81	0.21
L5_LBC	4.05	0.15	0.17	0.15	0.15	0.15	0.26	0.15	0.30	0.22	0.00	0.17
L5_MC	3.89	0.19	0.93	0.19	0.99	0.17	0.22	0.19	0.97	0.24	0.01	0.19
L5_NBC	3.60	0.17	0.20	0.17	0.27	0.18	0.51	0.17	0.29	0.21	0.31	0.19
L5_SBC	4.08	0.21	0.90	0.21	0.93	0.21	0.89	0.22	1.00	0.25	0.65	0.22
L5_TTPC1	3.58	0.12	0.00	0.13	0.06	0.14	0.15	0.14	0.21	0.20	0.00	0.15
L5_TTPC2	3.56	0.13	0.08	0.14	0.41	0.14	0.32	0.15	0.76	0.22	0.00	0.15
L5_UTPC	3.36	0.16	0.00	0.20	0.13	0.20	0.51	0.20	0.37	0.20	0.22	0.21
L6_BPC	2.76	0.13	0.00	0.18	0.32	0.19	0.63	0.18	0.45	0.15	0.00	0.19
L6_DBC	2.74	0.06	0.62	0.06	0.55	0.07	0.69	0.06	0.61	0.13	0.53	0.08
L6_LBC	2.86	0.14	0.78	0.13	0.74	0.13	0.64	0.14	0.88	0.18	0.11	0.14
L6_MC	2.97	0.13	0.25	0.13	0.18	0.14	0.35	0.13	0.26	0.16	0.59	0.15
L6_NBC	2.51	0.11	0.14	0.11	0.12	0.12	0.21	0.12	0.11	0.12	0.20	0.16
L6_NGC	2.99	0.21	0.00	0.43	1.00	0.35	0.56	0.44	0.95	0.19	0.00	0.43
L6_SBC	2.88	0.17	0.63	0.18	0.67	0.18	0.83	0.17	0.58	0.16	0.32	0.19
L6_UTPC	2.40	0.15	0.00	0.19	0.65	0.20	0.79	0.20	0.92	0.15	0.00	0.20

Mean bouton densities in experimentally characterized m-types, based on all potential synapses (“All”) and after application of the pruning algorithm (“Pruned”) for different touch distances. P-values as in Table 1.

Even though *C*_*p*_ was not used as a constraint, the strong correlations with biological values remained, even after completing all three pruning steps (Figure 6C, *r* = 0.71, *p* < 0.01, *N* = 14; see also Table 2). The only exceptions involved connections from Martinotti Cells (MCs) in layer 5, where the algorithm did not predict the high values (>0.3) reported by previous studies (Silberberg and Markram, 2007), and MC to PC connections in layer 2/3, for which some reports have suggested a *C*_*p*_ close to 1 (Fino and Yuste, 2011). However, Equation I shows that stronger connectivity would require an increase in axonal arborization or a major reduction in density of L5 PCs. Since the cell densities are well validated (see Markram et al., 2015), it is possible that previous reconstructions of the axonal arborizations of MCs were incomplete (but see below).

### Validation of emergent microcircuit features

**Figure 7:**
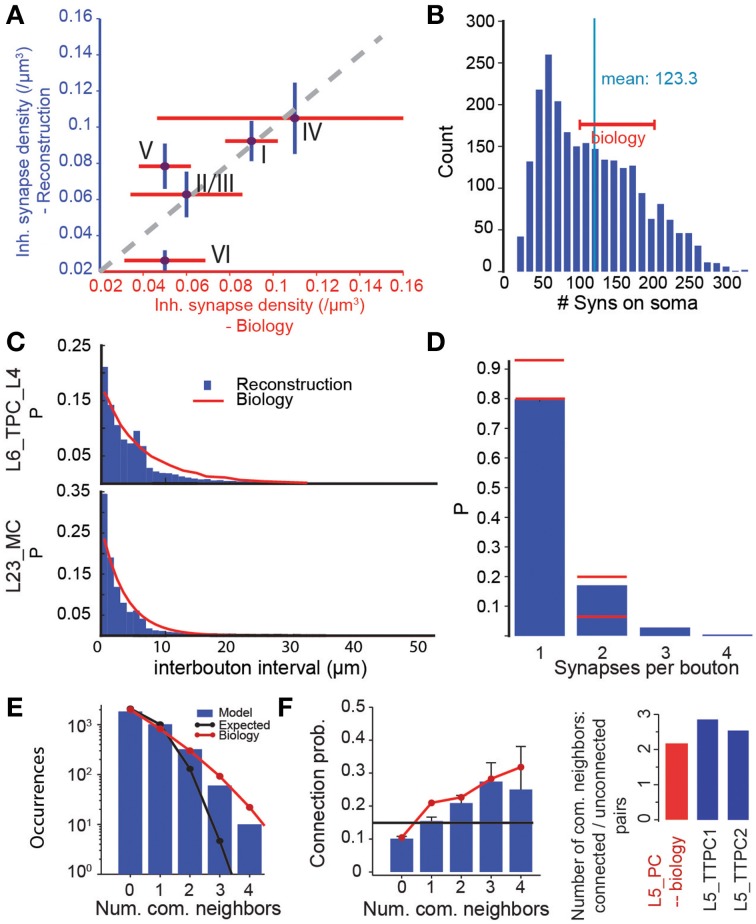
**Validation of emergent properties. (A)** Volumetric density of inhibitory synapses at the centers of the layers compared to measurements from electron microscopy. Purple circles indicate means, red lines the SD of the EM data (DeFelipe, personal communication), blue lines the SD of the reconstruction. **(B)** Distribution of the number of inhibitory synapses on the somas of L5_TTPC1 and L5_TTPC2 cells. Red bar: experimental data (DeFelipe, personal communication). **(C)** Distribution of intervals between efferent synapses in the model (blue bars) and biological inter-bouton intervals (red lines) for two m-types. Biological data from (Karube et al., 2004) (L23_MC) and (Anderson et al., 2002) (L6_PC). **(D)** Distribution of the number of synapses per bouton under the assumption that efferent synapses with an unbiologically low interval between them (<1 μm) were formed by the same bouton. Red lines: Data from (Bopp et al., 2014, mouse) and (DeFelipe, personal communication). **(E)** Distribution of the number of common neighbors of pairs of L5_TTPCs. Blue bars: Data from the reconstructed connectome obtained in an *in silico* patch experiment (see Methods). Red line: Data from (Perin et al., 2011). Black line: Data expected in a network with uniformly and independently random connectivity. **(F)** Left: Unidirectional connection probability between L5_TTPCs with different numbers of common neighbors. Blue bars, red line, black line as in **(E)**. Right: The ratio of the mean number of common neighbors of connected and unconnected pairs of L5_PCs resulting from the data in the left panel. Blue: data for the two types of L5_TTPCs in the reconstructed connectome, red: (Data from Perin et al., 2011).

To validate the predicted micro-scale connectome as a whole, we compared some of its emergent anatomical properties to biological data not used in the reconstruction. We found that, overall, it accurately reproduced the layer-specific densities of GABAergic synapses determined in an earlier EM study (DeFelipe, personal communication) (Figure 7A, *r* = 0.79, p = 0.11, N = 5). However, the predictions for L5 to L6 were significantly different. In the reconstructed connectome, L5 pyramidal somata were characterized by an average of 123 GABAergic synapses (Figure 7B), a value that matched the range of 100–200 found in a 3D confocal microscopy study of the perisomatic GABAergic (vGAT) innervation of the same m-type (DeFelipe, personal communication). Taken together, these validation tests suggest that the reconstructed connectome provides a reasonable reproduction of the overall and layer-specific level of inhibitory synapses in the microcircuit. They also suggest that a major increase in the size of the MC axonal arborization to increase the connection probability onto PCs is not possible, since it would also increase the overall density of GABAergic synapses (**see** Figure 6C). Another way of increasing MC to PC connection probabilities would be to make MCs target only PCs. Given, however, that MCs already form 90% of their synapses on PCs, this is not a viable solution (see Figure S2). Taken together, these results suggest that it may be necessary to revisit the experimental data on MC to PC connection probabilities.

The algorithm not only reproduced bouton densities as described above (Figure 5B), but also reported *distributions* of boutons, although they were not used by the algorithm (Figure 7C). This match to biological data supports the notion that a statistical approach to connectivity is valid. However, we found that, when a single axon in the reconstruction formed two synapses, the interval between them was often shorter than 1 μm (Figure 7C). This contradicted previous reports that such biological axons do not display such short intervals (Anderson et al., 2002; Karube et al., 2004), and implied that the same bouton may form synapses onto two different postsynaptic neurons. To test this possibility, we counted the number of occurrences of multiple synapses on the same bouton (interval between synapses < 1μm), and compared the results to data from EM studies (DeFelipe, personal communication). In both cases, ~20% of boutons formed multiple synapses (Figure 7D; although Bopp et al. (2014) report fewer multiple synapses in mouse). This further supports the statistical nature of connectivity.

To test the emergence of complex connectivity, we conducted *in silico* 12 patch-clamp experiments, in which recorded neurons occupied the same relative positions as those used in a previous *in vitro* study (Perin et al., 2011; see Methods), and identified all synaptically coupled pairs. The *in silico* experiments found the same distribution of numbers of common neighbors found *in vitro* (Figure 7E). As in the *in vitro* study, we also found a significant dependency between connection probabilities for pairs of neuron, and the number of their common neighbors (Figure 7F, left). Taken together, these data indicate a degree of clustering among synaptically connected neurons, similar to the clustering observed in biology (Figure 7F, right).

### Robustness of the predictive connectivity

**Figure 8:**
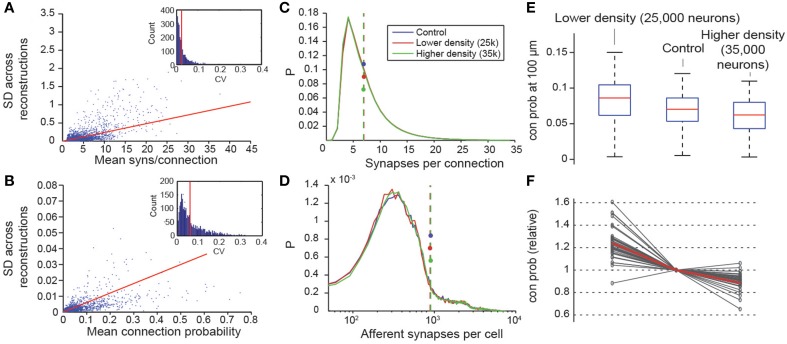
**Variability and robustness of emergent connectivity**. **(A)** Mean number of synapses per connection for all connection types, against the standard deviation of the mean across *N* = 7 reconstructions. Blue dots: data, red line: linear fit. Inset: Distribution of the coefficient of variation of the measurements of the mean, i.e., SD divided by mean. Red line: Mean CV. **(B)** Same, for the mean connection probability of individual connection type. **(C)** Distributions of the number of synapses in all connections for different neuron densities. Blue: microcircuit containing 31,000 neurons (control case); red: Only 25,000 neurons in the same volume; green: 35,000 neurons in the same volume. Dashed lines with disks indicate the mean. **(D)** Same for the number of afferent synapses per cell. **(E)** Box plot of the probabilities that a neuron connects to a randomly picked cell within 100 μm, for different neuron densities. Red lines indicate the median of the probabilities for all cells, blue boxes the 25 and 75 percentiles and black whiskers the full data spread. **(F)** Means of the connection probabilities for all m-types, normalized to the value of the control case (gray lines). The red line indicates the values expected if the changes in cell density were compensated exclusively by changes in connection probabilities as outlined in Equation (I) and Figure 1, i.e., 31,000/25,000, 1 and 31,000/35,000.

We have shown that, when the micro-scale connectome algorithm is constrained with biological data, it accurately recreates many features of biological connectivity, and that when it is constrained with predicted properties of the microcircuit, it is slightly less accurate. The derived connectome allowed us to make verifiable predictions for m-type to m-type connections that have not yet been measured experimentally. To assess the precision of the predictions, we evaluated the internal variability of the results generated by the algorithm. Seven microcircuits were generated from the same pool of reconstructed morphologies, using different exemplar morphologies in different random positions for each instance (see also Markram et al., 2015). As a measure of variability, we calculated the standard deviations of the *C*_*p*_ at a distance between somata of 100 μm (Figure 8B), and the mean numbers of synapses per connection for all seven microcircuits (Figure 8A). We found that, on average, the 95% confidence interval was around 5% of the mean value for the connection probabilities and smaller than 2% of the mean value for synapse numbers (see Methods). This suggests a precision of roughly ±2% of the mean, when biological properties are known, and roughly ±5% when they are not.

Two constants were crucial for determining the connectome: cell densities and total axonal length (see Markram et al., 2015; Figure 1A). Values for these constants were derived from the digitally reconstructed microcircuit prior to any pruning. To assess the impact of potential inaccuracies in cell densities, we constructed two additional microcircuits with lower and higher cell densities, using the approach in which all parameters were derived. The first microcircuit contained 25,000 neurons and the second, 35,000. In both cases, the distribution of synapses per connection was maintained (Figure 8C), as was the total number of synapses per neuron (Figure 8D). The reconstructions confirmed that connection probabilities were higher when cell density was lower, and lower when cell density was higher, as predicted by Equation I (Figures 8E,F). The equation also made it possible to predict the size of these changes (Figure 8F; gray lines, measured; red line, calculated).

## Discussion

We have isolated a set of fundamental principles and properties of synaptic connectivity that govern the organization of the local connectome, and used them in an algorithm that reconstructs the micro-scale connectome of a 3D digitally reconstructed microcircuit. Potential synapses were derived from the incidental appositions between exemplars of morphologically reconstructed neurons, placed randomly within their layer in such a way as to respect layer-specific neuron densities and neuron numbers. We found a relationship between synapse and apposition numbers per connection, which allows prediction of synaptic connectivity from appositions. By randomly removing potential synapses that cannot form actual synapses, for functional reasons, we arrived at a subset of biologically viable synapse locations. However, this subset was still far larger than the number of synapses observed in nature. We showed that simple statistical pruning of potential synapses does not reproduce biological connectivity, but that a three-step pruning process—general, multi-synapse and plasticity pruning—does. We further showed that the minimal biological data set required to reconstruct the local connectome of a given microcircuit of neurons consists of the mean bouton densities and the relation between the mean number of potential synapses per connection and the mean number of actual synapses per connection for as many m-types as possible. The algorithm reproduces a spectrum of features observed in actual neocortical microcircuitry, which vary by less than ±5% across different statistical instantiations of the microcircuit.

### Implications for the formation of synaptic connections

The algorithm determined synapse locations statistically, using 3D neuronal reconstructions and applying basic principles and properties of synaptic connectivity. The few cases where this was not possible suggest that experimental data on these exceptions could further improve the accuracy of the predicted connectome. The rule that several synapses are required to form a viable synaptic connection suggests that synapses act synergistically to ensure the survival of connections—a fundamental *synapse co-dependency mechanism*. This suggestion is supported by reports that synaptic connections in the neocortex and many other brain regions display heterosynaptic plasticity (Bonhoeffer et al., 1989; White et al., 1990; Schuman and Madison, 1994; Royer and Paré, 2003). This may be a result of energy constraints that limit the maximal number of synapses formed by an axon. Selection of the set of active multi-synapse connections is likely to depend on other types of microcircuit plasticity. One candidate could be a spike-timing-dependent plasticity (STDP) rule that includes re-wiring.

The finding that the minimal number of potential synapses between a pair of cells required for the formation of a synaptic connection differs between m-type specific pathways points to a variable level of synapse co-dependency for different m-type to m-type connections. The algorithm also predicts that if *S*_*m*_ is decreased, *C*_*p*_ will increase proportionally (if other fundamental parameters remain constant). Thus, the level of synapse co-dependency also determines the number of neurons that any one neuron can target.

### Bridging the gap to form a synapse

The algorithm pruned potential synapses to predict actual synapses. When potential synapses were defined by a maximum touch distance of 1.25 μm (i.e., all near touches closer than 1.25 μm) on m-types known to form spines, the algorithm successfully reproduced biological connectivity for many, but not all m-type to m-type connections. With a touch distance of 2.5 μm, the algorithm reproduced biological observations for all connection types that have been characterized experimentally, achieving a reasonable level of accuracy. The touch distances required to reproduce biological connectivity are compatible with biological observations. More than 10% of spine necks are longer than 1.25 μm (Arellano et al., 2007) and including the spine head, around 50% of spines extend beyond 1.25 μm, with a mean length of 1.3 μm and a maximum of 5.1 μm (although 95% are shorter than 2.6 μm; Benavides-Piccione, DeFelipe, unpublished observations). Boutons bridge an additional 0.25 μm. The algorithm therefore predicts that appositions 2.5 μm or below are biologically plausible locations for synapse formation. Since the number of potential synapses in a connection is invariably higher than the number of actual synapses, the fraction that become actual synapses may be set, in part, by the probability that a potential synapse closes this gap successfully.

### Deviations from experimentally measured connectivity

Since connection probabilities are strongly dependent on the distance between neuronal somata, and previous studies seldom account for distance-dependent connection probabilities, it was not possible to design a reliable algorithm based on these data. Interestingly, an *in silico* patch experiment, using the same sampling techniques as *in vitro* studies, predicted the same distant-dependent *C*_*p*_ (see Methods; Figure 4D), yet when all relevant cell pairs were measured the results deviated significantly (Figure 6C, squares vs. diamonds). The algorithm therefore does not rely on *C*_*p*_, which is instead an emergent, hence predicted property. Considering these limitations, the predicted *C*_*p*_ values matched experimental data reasonably well. However, the value for the L5_MC to L5_TTPC pathways was underestimated (Figure 6C). The reason for this mismatch remains unclear, but the experimental data for *C*_*p*_ for this pathway from different labs are also incompatible (Silberberg and Markram, 2007; Fino and Yuste, 2011).

The inter-dependencies among circuit properties, outlined in Figure 1A, imply that increasing *C*_*p*_ beyond the predicted values would require extreme changes to the other parameters. For example, it would require halving *C*_*d*_ or *S*_*m*_, or doubling *B*_*d*_ or *A*_*l*_ (or a combination thereof). *C*_*d*_ for PCs is supported extensively by experimental data and validated in various ways (see Figure S1A; Markram et al., 2015). It is therefore unlikely that this is the source of the deviation.

To test the possibility that deviations were due to inadequate reconstructions of axonal arborizations (i.e., that *A*_*l*_ was too low), we compared the density of inhibitory synapses in the reconstruction against biological estimates. Since inhibitory synapses in the microcircuit are formed by local interneurons, we expected that they would reach their full density in a microcircuit fully surrounded by six other microcircuits (Figure 7A). We found that in this configuration, the predicted density of inhibitory synapses was indeed highly correlated with data from EM, especially in layer 5, where the underestimation of *C*_*p*_ is apparently the greatest. Increasing *A*_*l*_ for the axons of Martinotti cells sufficiently to reproduce the reported *C*_*p*_ (i.e., doubling its value) would increase the predicted density of inhibitory synapses well above reported values.

We also considered another possible explanation, namely that Martinotti cells are entirely selective, using all their boutons to form synapses onto pyramidal cells. Given, however, that even in the current configuration, more than 90% of L5_MC synapses form on pyramidal cells (Figure S2), 100% specificity would produce only a minor increase in *C*_*p*_. It is also possible that MC connections to PCs are specifically oriented toward each other. There is no evidence for orientation in the horizontal plane. However, if MCs are consistently positioned just below or above PCs (Kozloski et al., 2001), this could explain the observed deviation in *C*_*p*_.

A final possibility is that biological values for *S*_*m*_ are overestimated. This possibility is supported by the fact that experimental values for *S*_*m*_ are based on light microscopic analysis and the only data validated by EM are for PC to PC connections. This example illustrates how deviations between the predictions of the algorithm and biological data can be used to challenge biological observations and their interpretations, or the assumptions used in the design of the algorithm (see Supplementary Table 1).

As more methods for large-scale experimental reconstruction of synaptic connectivity are developed and employed—for example EM with automated or semi-automated reconstruction techniques (Denk and Horstmann, 2004; Chklovskii et al., 2010; Kleinfeld et al., 2011)—we expect more deviations of the predictions of the algorithm to be found. They will be a valuable source of information for further refinement of the algorithm, for example providing additional data points for *S*_*m*_ or additional exceptions to the synapse location rule.

### A large potential for information storage

After multi-synapse pruning, we found that there was still an excess of potential synapses (*B*_*d*_ values higher than reported). This excess guided step 3 (removal of multi-synapse connections), and thereby predicted the potential of a connection for rewiring—its plasticity potential. The formation and elimination of multi-synapse connections during rewiring has been demonstrated experimentally (Le Be and Markram, 2006). In the algorithm, rewiring potential is represented by the third parameter of the algorithm, *a*_3_, whose value, for a touch distance of 2.5 μm, lies in the range 0.2–0.3 for excitatory connections, and 0.1–0.25 for inhibitory connections. For all connection types, touch distances ≥2.5μm yielded values ≤ 0.5 compatible with the doubling of connection probabilities under stimulation, observed experimentally (Le Be and Markram, 2006), These values allow a first approximation of the information storage capacity of the microcircuit. For example, values of *a*_3_ = 1 or *a*_3_ = 0 would leave no degrees of freedom for the selection of active connections, since either all would be active, or all would be inactive. The information contained in a given wiring diagram and the mean hamming distance between wiring diagrams are both maximal for *a*_3_ = 0.5. At *a*_3_ = 0.3 it is still possible to reach 88% of the maximum, while at 0.1 it is only possible to achieve 50%. This indicates the presence of substantial potential for plasticity, with significantly larger potential in excitatory than in inhibitory connections.

## Conclusion

The algorithm presented here provides a method for predicting the micro-scale connectome from sparse experimental data. When all afferents from beyond the microcircuit are accounted for (e.g., in reconstructions of the whole brain), it will be possible to use synapse density on the dendrites (i.e., complete utilization of dendrites) as an additional constraint, further improving the predictions. Since the predictions will fail if data on neuronal composition and morphologies are incorrect, the algorithm can be used to test experimental data. In this way, the predicted micro-scale connectome can complement future experimental work, accelerating progress toward a complete mapping of the connectome.

## Methods

### Pruning potential synapses

The modeled volume of neural tissue contained a large number of axo-dendritic, axo-somatic and axo-axonic appositions that we considered as locations of potential synapses. A preparatory filtering step eliminated all potential synapses, except those that were located on biologically plausible parts of the postsynaptic cell: dendrites in the case of pyramidal to pyramidal connections (Somogyi et al., 1998; Feldmeyer et al., 2002; Kawaguchi et al., 2006; Kubota et al., 2007); the axon initial segment for connections in the case of chandelier cells (ChCs) (Somogyi, 1977; Somogyi et al., 1982; Howard et al., 2005; Szabadics et al., 2006) and dendrite or axon for others.

To derive a biologically plausible connectome, we employed a three step pruning algorithm, a modified version of the algorithm proposed in (Fares and Stepanyants, 2009):

In the first step—*general pruning*—for each synapse we drew an independent random number *R* in the interval [0,1) and compared it against a parameter *f*_1_.

If *R* < *f*_1_ the potential synapse was admitted to the second step or else kept inactive in a pool accessible to structural plasticity mechanisms.

In the second step—*multi-synapse pruning*—we drew random numbers *R* ∈ [0, 1] for every connection. A connection was defined as the set of all potential synapses between a pre- and a postsynaptic cell. The connection was admitted to the next step only if

(M-I) $$\begin{array}{l} R<{\left(1\;+\;e^{-16∕\mu_{2}\cdot \left(N_{s}-\mu_{2}\right)}\right)}^{-1}, \end{array}$$

where *N*_*s*_ was the number of potential synapses forming the connection, and μ_2_ a parameter to this second step. Thus, the probability of admitting a connection is a rising sigmoidal function of the number of potential synapses contributing to the connection. In this simplified version of the criterion described by (Fares and Stepanyants, 2009), the width of the transition of the sigmoidal is set to its offset from the origin (here: μ_2_) multiplied by 0.25. In the results presented in (Fares and Stepanyants, 2009), the 95% confidence region for this parameter was very wide compared to that of the offset. This suggests that this parameter is relatively unimportant for achieving a good fit to the biological data. The value of 0.25 used to calculate the width of the transition was chosen to ensure that the fraction of connections with only one synapse was < 1%.

In the third step—*plasticity pruning*—whole connections were again removed randomly and independently. This time, however, potential connections were converted into active connections whenever *R* < *a*_3_ (*a*_3_ being the parameter of the third step), guaranteeing that connection pruning was independent of the number of potential synapses. Connections removed during this process were placed in a pool of *viable multi-synaptic connections* for future use by structural plasticity mechanisms.

### Finding parameters for the pruning algorithm

The pruning algorithm required three parameters for each individual pathway, i.e., for each combination of pre- and postsynaptic m-type. Depending on the pathway, we employed one of two methods to find a suitable combination of parameters that differed in which properties of connectivity we tried to match. For 38 pathways that have been reliably characterized experimentally (see Table 1), we tried to match the experimentally measured mean and standard deviation of the distribution of synapses per connection. For all other pathways (*N* = 2987), we first predicted the mean number of synapses per connection from the mean number of potential synapses (i.e., appositions) per connection (1.5·*S*^*struc, m*^ for E-E pathways, $9\;\cdot \sqrt{S^{struc,m}-1}-2$ for all other pathways). Next, we combined a generalized coefficient of variation of the distribution of synapses per connection of 0.32 with its predicted mean to predict its standard deviation. In both cases, we used the total number of efferent synapses on the presynaptic morphology type, derived from biological mean bouton densities multiplied by axon lengths, to further constrain the parameter space. Specifically, we used the same target fraction of potential synapses to be kept for each connection type of the same presynaptic morphology type, i.e., we did not assume any connection specificity beyond the specificity already present in the potential synapses.

Both strategies fully constrained the parameters. The way biological or generalized constraints lead to algorithm parameters is described in detail in the Supplementary Methods, and illustrated in Figure S1. Briefly, we quantified the impact of the parameters on the connectivity metrics in the derived connectome analytically, based on the finding that the number of potential synapses per connection follows a geometric distribution. Mathematically, the problem turns into an equation system with the three parameters as unknowns, meaning that three constraints are needed to find a unique solution. Of the metrics and parameters introduced in Figure S1, any combination of three leads to a unique solution.

To derive the connectome, we combined three constraints: *S*_*m*_, *S*_*sd*_, *B*_*d*_. Solving the set of equations (see Supplementary Methods), led to the following equations:

(M-II) $$\begin{array}{l} f_{1}=\frac{p}{1-p}\cdot \frac{1-p^{\text{′}}}{p^{\text{′}}}, \end{array}$$

where *p* is the inverse of the initial number of potential synapses per connection in the input and $p^{{}^{\prime }}=\frac{1}{S_{sd}+0.5}$ is the inverse of the mean number of potential synapses per connection after the first step. Then:

(M-III) $$\begin{array}{r} \mu_{2}=0.5+S_{m}-S_{sd} \end{array}$$

(M-IV) $$\begin{array}{r} a_{3}=\frac{B_{d}}{B^{2}}, \end{array}$$

where *B*^2^ is the bouton density after the first two pruning steps. In our derivation of the connectome, we used these equations to determine parameter values.

### Calculation of bouton densities

Since the density of fibers decreased near the boundaries of the volume of the digital reconstruction, we calculated bouton densities in the most central region, where densities have been shown to match biological levels (see Figure 2, Markram et al., 2015). For the calculation of volumetric bouton densities per layer, we selected four 25 × 25 × 25 μm volumes at the centers of each layer. The volumes were offset by [–12.5 μm, –12.5 μm], [–12.5 μm, 12.5 μm], [12.5 μm, –12.5 μm], and [12.5 μm, 12.5 μm] in the x/z directions from the geometrical center of the layers, resulting in four non-overlapping volumes. Densities for individual volumes were computed by counting the number of (GABAergic) synapses it contained, and dividing by the volume. Synapse counting in these volumes used spatial indexing software as described in Tauheed et al. (2012). When calculating the density of synapses along axons and intervals between neighboring boutons, we only considered synapses and parts of axons less than 37.5 μm from the central y-axis of the volume of the digital reconstruction (i.e., the analyzed volume had roughly the same size as the one analyzed for the volumetric densities). Efferent synapses separated by less than 1 μm on the same axon were considered as sharing a bouton.

### *in silico* patch clamp sampling

To sample the connectivity of neuron pairs, we recreated a previous *in vitro* patch clamp experiment (Perin et al., 2011). We placed the recorded relative coordinates of simultaneously patched somata at a random location within a target layer of the digital reconstruction. For each resulting coordinate, we found the closest neuron of a morphological type of interest. We then analyzed the connectivity of the resulting sets of neurons, probing up to 12 neurons and 132 connections simultaneously. We used 46 sets of patch coordinates with 8.2 ± 3.4 coordinates each.

### Conflict of interest statement

The authors declare that the research was conducted in the absence of any commercial or financial relationships that could be construed as a potential conflict of interest.
