# Supplementary material for: An algorithm to predict the connectome of neural microcircuits
Source: Front Comput Neurosci. 2015 Oct 8;9:120. doi: 10.3389/fncom.2015.00120 (PMC4597796; doi:10.3389/fncom.2015.00120)
Supplement: Supplementary file 1 [file SupplementaryTable1.PDF]

| Assumption | Potential consequences if incorrect | Justification |
|---|---|---|
| ***Cell densities and placement (neuronal composition)*** | | |
| *Connectome derivation is based on the assumption that neurons are placed in the model at biologically accurate locations and densities. The methods for neuron density measurements and neuron placement are outlined in Markram et al., submitted.* | As shown in Figure 8, cell densities directly influence the connection probabilities. Inaccurate placement of dendritic and/or axonal arborizations, especially relative to layer boundaries, could lead to a violation of the *synapse location rule* and to inaccurate strength of trans-laminar pathways. | Validations of cell densities and placement are described in Markram et al., submitted . |
| ***Morphological reconstructions*** | | |
| *It is further assumed that the morphological reconstructions are accurately classified and arbors are complete, at least locally, i.e. the part that falls within the modelled volume. Reconstruction and repair procedures are outlined in Markram et al., submitted.* | Locally incomplete reconstructions would lead to underestimation of connection probabilities. | Validations of the reconstructions are described in Markram et al., submitted and in Figure 2. |
| ***Uniformity of bouton density for a given morphological type*** | | |
| *In addition to the overall distribution of inter-bouton intervals matched by the model, there may be systematic variability of bouton densities. For example, varying with axonal branch order or relative to layers.* | Locally increased / decreased bouton density would increase / decrease connection probabilities in that region. | Exceptions will have to be added in the future. |
| ***Distribution of synapses per connection sufficiently described by mean and standard deviation*** | | |
| *While we attempt to match the known mean and standard deviations of connection types, the exact shape of that distribution is not explicitly matched but determined by the following: The right side remains in the shape of a geometric distribution after general pruning, the left side is shaped by a sigmoidal with a transition width that is ¼ of its offset.* | The number of synapses in a connection influence the strength and reliability of a connection.<br><br>Yet, as long as the mean matches, the emerging connection probabilities will be correct (Equation I). | As the number of connected neuron pairs sampled sampled in the literature is typically low, the true shape of the distribution can hardly be determined. With the current method, results of the algorithm are statistically indistinguishable from the reported data. This may change as more data gets reported. |
| ***Distribution of appositions on dendrites and axon yield biological synapse distributions*** | | |
| *The "synapse location rule".* | Synapse locations on the dendrite determine the degree of attenuation of PSPs and the possibility of local interaction between synaptic conductances (for example shunting inhibition). | The rule has been shown to be valid for a number of connection types by Hill et al., 2011. |
| ***No axonal selectivity beyond what is expressed by algorithm parameters and exceptions to the synapse location rule*** | | |
| *Potentially selective formation of synapses by axons is implemented in the algorithm mainly by having one set of parameters per connection type. For example, a high value of $\mu_2$ leads to selective avoidance of that connection type. In addition, two explicit exceptions are formulated (see Results).* | This can have large consequences on the predicted connectome. From significant strengthening of connection types to outright removal of others. | We expect more exceptions to be formulated in the future to be in line with experimental results. Experimental data on such exceptions will be extremely valuable for improving the predicted connectome. |
| ***Distribution of appositions per pair of neuron can be approximated by a geometric distribution and mathematical simplification during parameter finding*** | | |
| *The equations used for parameter finding start with the assumption that the distribution of appositions per connection can be approximated by a geometric distribution. In addition, two mathematical simplifications are made (see Supplementary Methods).* | May lead to slight inaccuracies in the resulting mean and standard deviation of synapses per connection. | An exact fit to mean values is not required, given the uncertainty due to low sample numbers in the reported data. It might even indicate overfitting. |
| ***Uniformity of axonal arbor within a layer*** | | |
| *Equation I takes the notion of integrating axonal density over space to – ultimately – arrive at a connection probability. This assumes that the axon is uniform within that space.* | The equation cannot be used to make quantitative predictions about connection probabilities. | Even if quantitative predictions cannot be made, the qualitative prediction – the number of connections with neurons in a region of space is bounded by the amount of axon and bouton density in that region – holds. |
| ***Mean number synapses per bouton 1.2*** | | |
| *It is assumed that the number of efferent synapses on a bouton has a mean of 1.2.* | This dictates how the bouton densities are translated into numbers of (efferent) synapses and therefore into numbers of connections and synapses per connection and ultimately connection probabilities. | The available biological data seems to indicate a value slightly above 1. |

Table S1 – Underlying modeling assumptions, potential consequences if assumptions are incorrect and justifications.
