# Supplementary material for: An algorithm to predict the connectome of neural microcircuits
Source: Front Comput Neurosci. 2015 Oct 8;9:120. doi: 10.3389/fncom.2015.00120 (PMC4597796; doi:10.3389/fncom.2015.00120)
Supplement: Supplementary file 2 [file SupplementaryTable2.PDF]

Layers	***Short name***	Full name
	**Inibitory**	
L1	**DAC**	Descending Axon Cell
L1	**NGC-DA**	Neurogliaform Cell with dense axonal arborization
L1	**NGC-SA**	Neurogliaform Cell with slender axonal arborization
L1	**HAC**	Horizontal Axon Cell
L1	**LAC**	Large Axon Cell
L1	**SAC**	Small Axon Cell
L23, L4, L5, L6	**MC**	Martinotti Cell
L23, L4, L5, L6	**BTC**	Bitufted Cell
L23, L4, L5, L6	**DBC**	Double Bouquet Cell
L23, L4, L5, L6	**BP**	Bipolar Cell
L23, L4, L5, L6	**NGC**	Neurogliaform Cell
L23, L4, L5, L6	**LBC**	Large Basket Cell
L23, L4, L5, L6	**NBC**	Nest Basket Cell
L23, L4, L5, L6	**SBC**	Small Basket Cell
L23, L4, L5, L6	**ChC**	Chandelier Cell
	**Excitatory**	
L23, L4	**PC**	Pyramidal Cell
L4	**SP**	Star Pyramidal Cell
L4	**SS**	Spiny Stellate Cell
L5	**TTPC1**	Thick-tufted Pyramidal Cell with a late bifurcating apical tuft
L5	**TTPC2**	Thick-tufted Pyramidal Cell with an early bifurcating apical tuft
L5, L6	**UTPC**	Untufted Pyramidal Cell
L5	**STPC**	Slender-tufted Pyramidal Cell
L6	**TPC_L4**	Tufted Pyramidal Cell with dendritic tuft terminating in layer 4
L6	**TPC_L1**	Tufted Pyramidal Cell with dendritic tuft terminating in layer 1
L6	**IPC**	Pyramidal Cell with inverted apical-like dendrites
L6	**BPC**	Pyramidal Cell with bipolar apical-like dendrites

Table S2 – Abbreviations of morphological types (m-types). A full m-type name includes a layer prefix from the first column and a short name from the second column.
