# Supplementary material for: An algorithm to predict the connectome of neural microcircuits
Source: Front Comput Neurosci. 2015 Oct 8;9:120. doi: 10.3389/fncom.2015.00120 (PMC4597796; doi:10.3389/fncom.2015.00120)
Supplement: Supplementary file 3 [file SupplementaryMethods.PDF]

Supplementary methods

We find parameters for the structural to functional conversion on a per-pathway basis. Instead of iteratively fitting the parameters to the available biological data, we analyzed the effect the parameters have on the connectivity to arrive at a predicted set of parameters. As the biological data come with comparatively low numbers of samples, an exact fit of for example mean synapse numbers is not required to yield results that are *statistically indistinguishable* from the biological data. As such we can use a number of mathematical approximations to simplify the analysis.

In the following section all experimental and model measurements as well as values for the parameters are meant to be considered for any single connection type, even when not explicitly mentioned.

Before conversion, we measure m_s, the mean of the distribution of structural appositions per connection and the density of efferent synapses on the presynaptic morphology type. Later measurement is combined with the target bouton density to yield B^*, the target filling fraction, i.e. the fraction of structural appositions converted to functional synapses.
In order to find the set of parameters – f_1, μ_2 and a_3 – that lead to the desired results, we consider that the distribution of appositions per connection has the shape of a geometric distribution:

$$P(n_s = x) = (1-p)^{x-1} \cdot p, \qquad \text{(S-I)}$$

for $p = \frac{1}{m_s}$ and $x \in \{1, 2, 3, \dots\}$
The variance of such a geometric distribution is $\frac{1-p}{p^2} = m_s{}^2 - m_s = (m_s - 0.5)^2 - 0.25$
In the following section, we use a simplification that discards the last summand, approximating the standard deviation as $\sqrt{(m_s - 0.5)^2} = m_s - 0.5$. The absolute error of the approximated standard deviation is < 0.09 for $m_s \geq 2$.

The first filtering step removes a fraction of $(1-f_1)$ synapses. This reduces the mean and standard deviation of the distribution.

$$\bar{n}_1 = \frac{1-p}{p} \cdot f_1 + 1 \qquad \text{(S-II)}$$

$$\sigma(n)_1 = \frac{1-p}{p} \cdot f_1 + 0.5 \qquad \text{(S-III)}$$

For the second step, first let p' be the shape parameter of the geometric distribution after step 1:

$$p' = \frac{1}{\bar{n}_1} = \frac{1}{\frac{1-p}{p} \cdot f_1 + 1}, P(n_1 = x) = (1-p')^{x-1} \cdot p' \qquad \text{(S-IV)}$$

The second step cuts off the left side of the distribution. To continue the analysis, we assume that all bins $\geq\mu_2$ are admitted and all bins $<\mu_2$ are removed, i.e. that the width of the transition of the sigmoidal function in multi-synapse pruning is infinitely narrow. In the actual pruning, a small number of connections $<\mu_2$ will be admitted and a small number of connections $\geq\mu_2$ will be pruned. We found that even with this approximation, our predictions of the resulting mean and standard deviation of the number of synapses per connection are accurate enough to yield results that are statistically indistinguishable from the biological data.

We see that under that assumption the cutoff is equivalent to translating the distribution by μ_2-1 to the right. The normalization factor for the distribution after the cutoff is equal to 1 minus the cumulative distribution of μ_2-1:

$$P(n_2 = x) = \frac{(1-p')^{x-1} \cdot p'}{(1-p')^{\mu_2-1}} = (1-p')^{x-(\mu_2-1)-1} \cdot p'$$

$$= P\big(n_1 = x - (\mu_2 - 1)\big), x \geq \mu_2 \qquad \text{(S-V)}$$

This increases the mean accordingly, keeping the standard deviation unchanged:

$$\overline{n}_2 = \frac{1-p}{p} \cdot f_1 + \mu_2 = \frac{1-p'}{p'} + \mu_2 \qquad \text{(S-VI)}$$

$$\sigma(n)_2 = \frac{1-p}{p} \cdot f_1 + 0.5 = \frac{1-p'}{p'} + 0.5 \qquad \text{(S-VII)}$$

This means for the coefficient of variation of the distribution:

$$cv(n)_2 = \frac{\sigma(n)_2}{\overline{n}_2} = \frac{\frac{1-p'}{p'} + 0.5}{\frac{1-p'}{p'} + \mu_2} \qquad \text{(S-VIII)}$$

$$\Leftrightarrow \mu_2 = \frac{\frac{1-p'}{p'} + 0.5}{cv(n)_2} - \frac{1-p'}{p'} \qquad \text{(S-IX)}$$

How many synapses are removed in the second step? To answer that, we first look at an alternative way to calculate the mean: The sum of each possible synapse number, multiplied with its probability:

$$\overline{n}_1 = \frac{1}{p'} = \sum_{i=1}^{\infty} (1-p')^{i-1} \cdot p' \cdot i \qquad \text{(S-X)}$$

Similarly, the formula for the mean of the distribution after applying the cutoff at μ_2:

(S-XI)

$$\Leftrightarrow (1-p')^{\mu_2-1} \cdot \left(\frac{1-p'}{p'} + \mu_2\right) = \sum_{i=\mu_2}^{\infty} (1-p')^{i-1} \cdot p' \cdot i \qquad \text{(S-XII)}$$

The factor before the sum serves as a normalization factor.
Finally, the fraction of synapses removed in the second step is:

$$R = p' \cdot \sum_{i=1}^{\mu_2-1} (1-p')^{i-1} \cdot p' \cdot i =$$

$$p' \cdot \left(\frac{1}{p'} - \sum_{i=\mu_2}^{\infty} (1-p')^{i-1} \cdot p' \cdot i\right) \qquad \text{(S-XIII)}$$

And inserting S-XII:

$$R = p' \cdot \left(\frac{1}{p'} - (1-p')^{\mu_2-1} \cdot \left(\frac{1-p'}{p'} + \mu_2\right)\right)$$

$$= 1 - p' \cdot (1-p')^{\mu_2-1} \cdot \left(\frac{1-p'}{p'} + \mu_2\right) \qquad \text{(S-XIV)}$$

$$= 1 - p' \cdot \mu_2 \cdot (1-p')^{\mu_2-1} - (1-p')^{\mu_2}$$

The factor p' before the sum normalizes it to a maximal value of 1 because the series converges to $\frac{1}{p'}$ (see equation S.X). Consequently, the fraction of appositions *remaining* after step 1 and 2:

$$B^{1,2} = f_1 \cdot (p' \cdot \mu_2 \cdot (1-p')^{\mu_2 - 1} + (1-p')^{\mu_2})$$

$$= \frac{p}{1-p} \cdot \frac{1-p'}{p'} \cdot (p' \cdot \mu_2 \cdot (1-p')^{\mu_2 - 1} + (1-p')^{\mu_2}) \quad \text{(S-XV)}$$

$$= \frac{p}{1-p} \cdot \left(\mu_2 \cdot (1-p')^{\mu_2} + \frac{(1-p')^{\mu_2+1}}{p'} \right)$$

We use the equations derived above to find a set of parameters using the following strategy.

Matching mean, standard deviation and bouton density

Let $\overline{n^*}$ and $\sigma(n)^*$ be the target mean and standard deviation. $p = \frac{1}{\overline{n_s}}$. Follow the following steps:

1. Calculate μ_2:

$$\mu_2 = 0.5 + \overline{n}^* - \sigma(n)^* \quad \text{(S-XVI)}$$

2. Calculate p':

$$p' = \frac{1}{\sigma(n)^* + 0.5} \quad \text{(S-XVII)}$$

3. Calculate f_1:

$$f_1 = \frac{p}{1-p} \cdot \frac{1-p'}{p'} \quad \text{(S-XVIII)}$$

4. Calculate an appropriate bouton reduction factor after step 1 and step 2 are completed:

$$a_3 = \frac{B^*}{B^{1,2}} \quad \text{(S-XIX)}$$
