# Supplementary material for: An algorithm to predict the connectome of neural microcircuits
Source: Front Comput Neurosci. 2015 Oct 8;9:120. doi: 10.3389/fncom.2015.00120 (PMC4597796; doi:10.3389/fncom.2015.00120)
Supplement: Supplementary file 4 [file SupplementaryFigureS1.PDF]

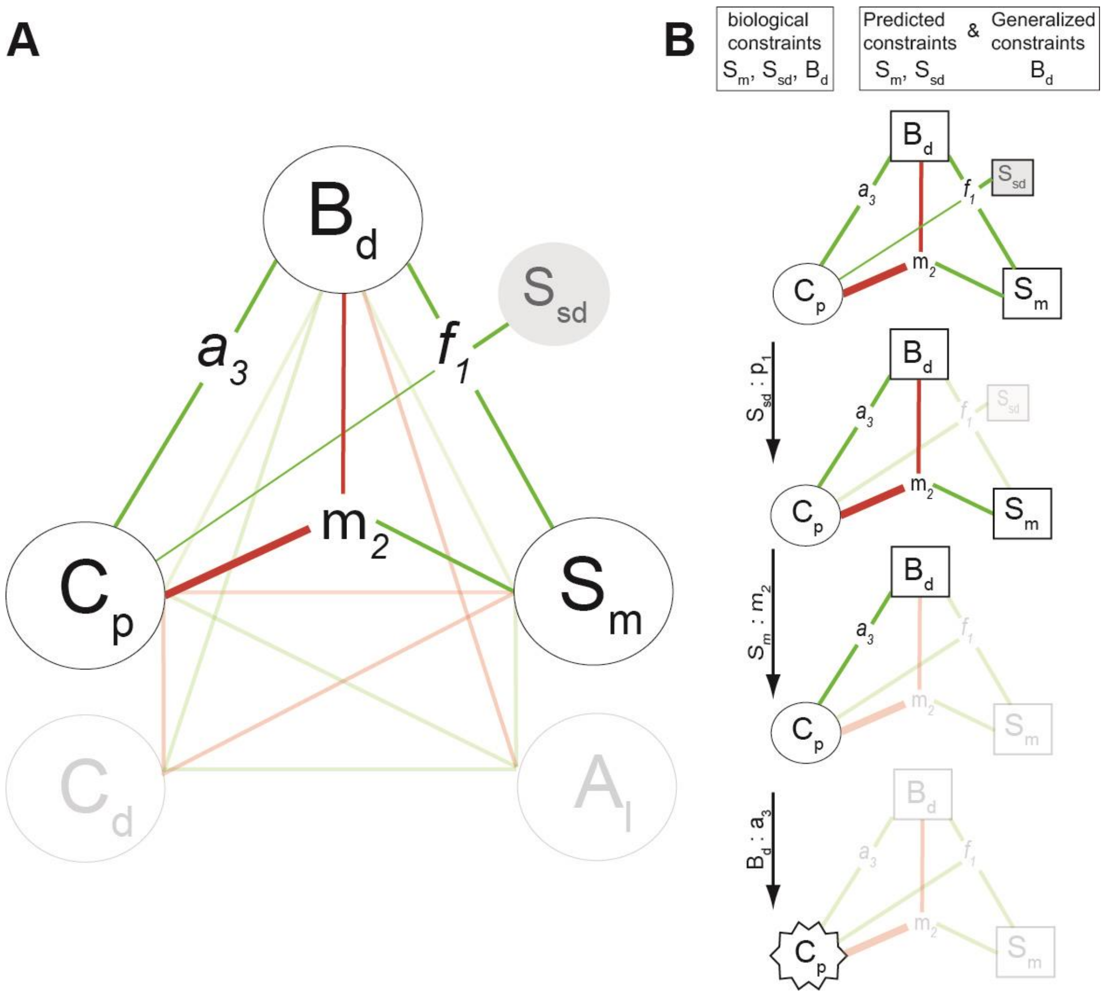

Figure S1: **Illustration of the parameter finding method**

(A) Dependency of metrics on the parameters of the pruning algorithm. Cell density and axonal length are experimentally provided and unaffected by pruning. Standard deviation of the number of synapses per connection (S_{sd}) is assumed and serves as an additional metric. The coefficient of variation (S_{cv}) is the ratio of standard deviation and mean. Green / red edges indicate that increasing the value of the parameter while keeping all else fixed, increases/decreases the metric. (B) Parameterization method: S_m, $S_s d$ and B_d are taken from the literature (biological parametrization) or extrapolated and generalized (generalized parametrization). As S_{sd} is determined only by f_1, we can match the biological value by choosing f_1. The only remaining parameter that determines S_m is μ_2. Finally, we can choose s_3 to match B_d, leaving C_p as a prediction.
