# Supplementary material for: An algorithm to predict the connectome of neural microcircuits
Source: Front Comput Neurosci. 2015 Oct 8;9:120. doi: 10.3389/fncom.2015.00120 (PMC4597796; doi:10.3389/fncom.2015.00120)
Supplement: Supplementary file 5 [file SupplementaryFigureS2.PDF]

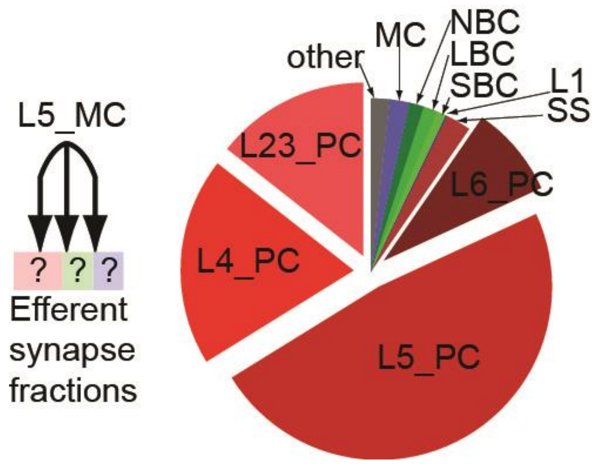

Figure S2: **Specificity of the L5_MC to PC pathways**

Specificity of L5_MC connectivity emerging from the pruning axonal appositions. The pie diagram depicts the fraction of efferent L5_MC synapses on individual postsynaptic m-types.
