# Supplementary material for: An algorithm to predict the connectome of neural microcircuits
Source: Front Comput Neurosci. 2015 Oct 8;9:120. doi: 10.3389/fncom.2015.00120 (PMC4597796; doi:10.3389/fncom.2015.00120)
Supplement: Supplementary file 6 [file SupplementaryFigureS3.PDF]

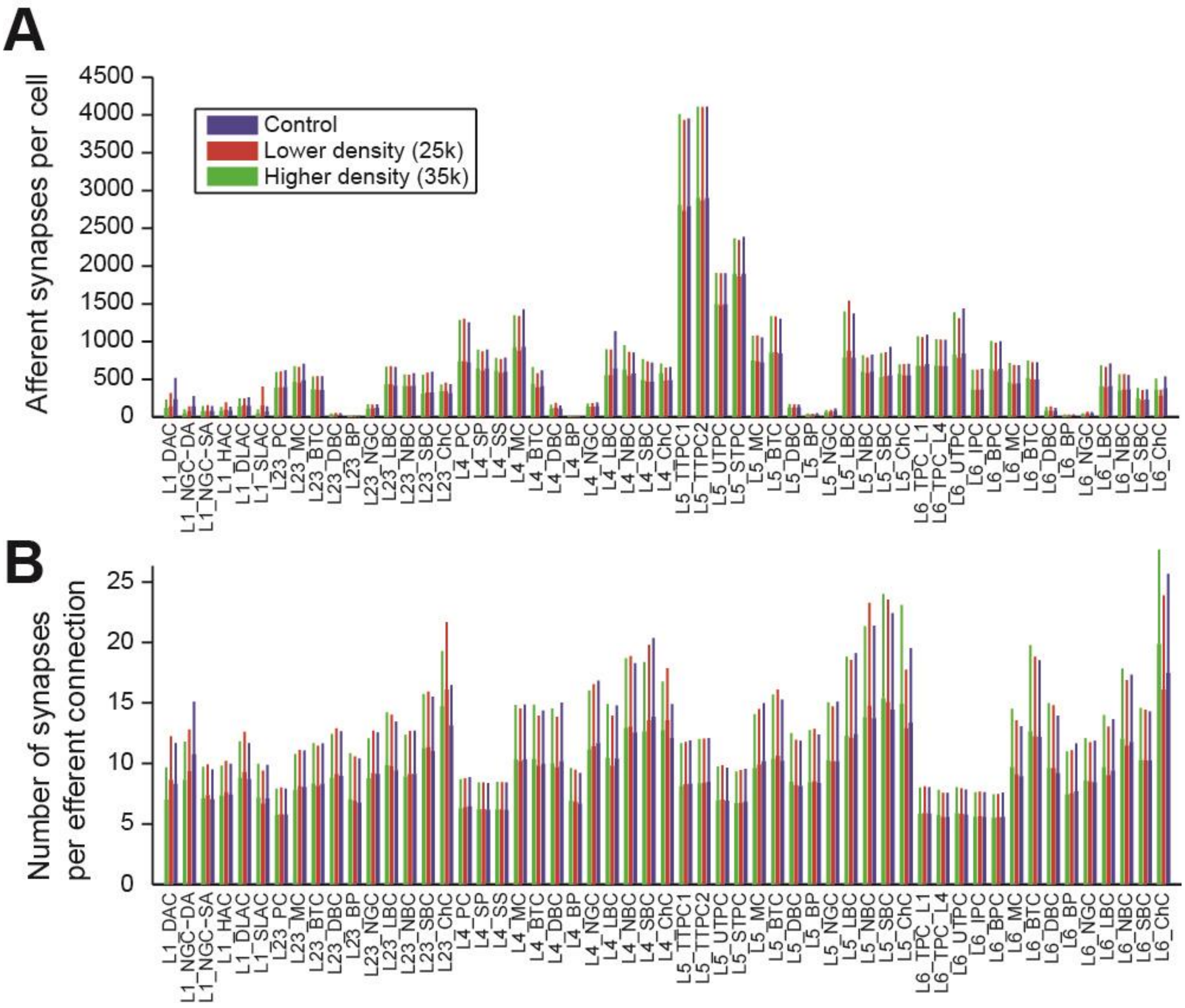

Figure S3: **Robustness of synapse counts against changes in cell density for individual pathways**

(A) Number of afferent synapses per neuron for all morphological neuron types. Bars indicate the mean, whiskers the standard deviation. Blue: 31,000 neurons in microcircuit (control); Red: 25,000 neurons in the same volume; green: 35,000 neurons in the same volume. (B) Same, for the number of synapses per efferent connection.
